# Low Folate and Selenium in the Mouse Maternal Diet Alters Liver Gene Expression Patterns in the Offspring after Weaning

**DOI:** 10.3390/nu7053370

**Published:** 2015-05-08

**Authors:** Matthew P.G. Barnett, Emma N. Bermingham, Wayne Young, Shalome A. Bassett, John E. Hesketh, Anabel Maciel-Dominguez, Warren C. McNabb, Nicole C. Roy

**Affiliations:** 1 Food Nutrition & Health Team, Food & Bio-Based Products Group, AgResearch Limited, Grasslands Research Centre, Tennent Drive, Palmerston North 4442, New Zealand; E-Mails: emma.bermingham@agresearch.co.nz (E.N.B.); wayne.young@agresearch.co.nz (W.Y.); shalome.bassett@agresearch.co.nz (S.A.B.); nicole.roy@agresearch.co.nz (N.C.R.); 2 Nutrigenomics New Zealand; Private Bag 92019, Auckland 1142, New Zealand; 3 Gravida: National Centre for Growth and Development, Private Bag 92019, Auckland 1142, New Zealand; 4 Institute for Cell and Molecular Biosciences and Human Nutrition Research Centre, Newcastle University, Newcastle upon Tyne, NE2 4HH, UK; E-Mails: j.e.hesketh@newcastle.ac.uk (J.E.H.); anabel.maciel@icloud.com (A.M.-D.); 5 AgResearch Limited, Grasslands Research Centre, Tennent Drive, Palmerston North 4442, New Zealand; E-Mail: warren.mcnabb@agresearch.co.nz; 6 Riddet Institute, Massey University, Tennent Drive, Palmerston North 4442, New Zealand

**Keywords:** microarray analysis, folate, selenium, high-fat diet

## Abstract

During pregnancy, selenium (Se) and folate requirements increase, with deficiencies linked to neural tube defects (folate) and DNA oxidation (Se). This study investigated the effect of a high-fat diet either supplemented with (diet H), or marginally deficient in (diet L), Se and folate. Pregnant female mice and their male offspring were assigned to one of four treatments: diet H during gestation, lactation and post-weaning; diet L during gestation, lactation and post-weaning; diet H during gestation and lactation but diet L fed to offspring post-weaning; or diet L during gestation and lactation followed by diet H fed to offspring post-weaning. Microarray and pathway analyses were performed using RNA from colon and liver of 12-week-old male offspring. Gene set enrichment analysis of liver gene expression showed that diet L affected several pathways including regulation of translation (protein biosynthesis), methyl group metabolism, and fatty acid metabolism; this effect was stronger when the diet was fed to mothers, rather than to offspring. No significant differences in individual gene expression were observed in colon but there were significant differences in cell cycle control pathways. In conclusion, a maternal low Se/folate diet during gestation and lactation has more effects on gene expression in offspring than the same diet fed to offspring post-weaning; low Se and folate *in utero* and during lactation thus has persistent metabolic effects in the offspring.

## 1. Introduction

Appropriate nutrition during pregnancy, in terms of both macronutrients and micronutrients, is important for normal embryonic and foetal growth. Limited maternal nutrition during this period increases neonatal mortality and reduces antioxidant enzyme expression leading to foetal brain oxidative stress and DNA damage [1,2]. In addition, this may result in cardiovascular defects, insulin resistance, and increased body fat in adulthood. There is also evidence that both energy dense and micronutrient-poor diets have a range of effects on the metabolism of the offspring during pregnancy and in later life [3]. In rats, for example, exposure of dams to a high-fat diet induced early reproductive maturation in the offspring who also developed obesity [4] and this effect may be compounded in subsequent generations exposed to a high-fat diet.

The so-called “Western” diet is typically macronutrient rich, containing high levels of fat, carbohydrate and protein; however, the intake of micronutrients may often be inadequate [5]. This is particularly observed in obese individuals where the prevalence of micronutrient deficiencies, such as that of the trace element selenium (Se) and the vitamin folate [6,7,8], is higher compared to normal weight individuals of the same age and sex [7,9,10]. 

The dietary micronutrient selenium (Se) is essential for human health [11] and for geological reasons in several parts of the world, including Europe and New Zealand, it is known that selenium intake is marginally low and sub-optimal for health in the adult population [11,12]. Unlike consumption of most other nutrients, selenium intake is not reliably assessed using self-report tools, such as food frequency questionnaires, because of the wide variations in soil selenium [13]. Selenium (Se) is incorporated into selenoproteins, several of which are involved in cellular antioxidant mechanisms [14]. It has been suggested that poor prenatal Se intake is linked to pregnancy disorders such as pre-eclampsia [15] and is detrimental for children’s psychomotor and language development [16]. Se also plays a role in reproduction, where it is essential for sperm production. In goats, maternal supplementation with Se during gestation and lactation influences the oxidative status and expression of apoptotic genes in the testes of offspring [17] while a recent rat study demonstrated that low Se levels were also associated with changes in intrauterine growth and liver metabolism [18]. 

The vitamin folate is a cofactor in several metabolic pathways, including methionine synthesis, glycine and serine inter-conversion, and purine and pyrimidine synthesis. Because of its important role in nucleic acid synthesis, sub-optimal folate levels during early gestation can impair cellular growth and replication in the developing foetus or placenta, leading to neural tube defects [19]. Furthermore, low concentrations of dietary and circulating folate during pregnancy are associated with increased risks of preterm delivery, infant low birth weight, and foetal growth retardation [19]. However, although awareness of the importance of folic acid has increased significantly over recent years, the number of women taking folic acid prior to conception remains very low [20,21] with global preconception use estimated to be less than 50% [22]. This is compounded by the fact that half of pregnancies are unplanned, and as a consequence, the point at which folic acid is most protective against neural tube defects (from preconception until the fourth week of pregnancy) may have occurred before the woman realizes she is pregnant [23]. Low folate intake during pregnancy has also been shown to lead to metabolic changes in the offspring post-weaning and to an altered metabolic response of the offspring to a high-fat diet after weaning [1,24]. 

Although high-fat diets are consumed in many countries where there is evidence of sub-optimal Se intake, the impact of marginally low Se and folate intake whilst eating a high fat diet during pregnancy and lactation on the metabolism of the offspring is not understood. We hypothesised that a combination of marginally deficient Se and low folate fed in a high fat diet during pregnancy and lactation would cause metabolic changes in the offspring post-weaning. In order to test this hypothesis, we carried out transcriptomic analysis of tissues from male mice born to dams fed a high-fat diet supplemented with Se/folate (diet H), or fed the same diet marginally deficient in Se/folate (diet L) during pregnancy and lactation. The offspring were subsequently fed one or other of these two diets post-weaning, resulting in four treatment groups (HH, HL, LL or LH). The Se content of the marginally deficient diet (0.08 mg/kg of diet) is comparable to that used previously (0.06 mg/kg; [25]) and was designed to provide an animal model for the sub-optimal levels of Se. Likewise, the level of folate in the marginally deficient diet (0.4 mg/kg of diet) is also comparable to that previously used in animal models [26,27]. We believe that these levels of dietary Se and folate are relevant to the sub-optimal, rather than frankly deficient, levels that may be found in human populations.

In this study, we focus on liver and colon because earlier work has shown that low Se affects liver metabolism *in utero* [18,25], and Se levels have also been reported to affect both intestinal growth in the early life of the lamb, and to influence susceptibility to colorectal cancer in the adult human [12,28,29]. The liver is an important Se reservoir and dietary Se and folate treatment together have an important effect on liver function resulting in increased Se deposits and balanced Se bioavailability [30,31]. This is especially important during gestation and lactation, and as a direct result, the health of the progeny is improved [31]. Similarly, the liver is also the main tissue responsible for folate storage and metabolism [32]. Supplementation with methyl donors including folate in a mouse model of colitis has been shown to affect gene expression and DNA methylation in the colon [33] implying that colon may be an important target tissue for folate supplementation [34]. Moreover, Se and folate together have an important effect on the colon where, together, high levels of Se and folate are associated with a substantially reduced risk of colon cancer [13]. However, little is known about how Se and folate interact. We have recently shown that high levels of Se and folate in the post-weaning diet of female offspring born to mouse dams fed a high-fat diet sub-optimal in Se/folate during gestation and lactation affected expression of genes involved in metabolism in both colon and liver [34]. Our aim in the current study was to investigate the effects of altered levels of Se and folate (supplemented *vs.* marginally deficient) in the same high-fat maternal diet during gestation and lactation, on male offspring using a double-crossover experiment.

Here we report that the metabolic impact of the low Se/folate diet on the offspring is considerably greater when fed to dams during gestation and lactation rather than to the offspring post-weaning, which suggests that these effects persist in the offspring throughout later life.

## 2. Experimental Section

### 2.1. Animal Experiments

This study was carried out in accordance with the recommendations of the New Zealand Animal Welfare Act 1999. The experimental procedures for this study were reviewed and approved by the AgResearch Grasslands Animal Ethics Committee in Palmerston North, New Zealand (Ethics Application No. 11691). All efforts were made to minimise animal suffering.

A pelleted sucrose-casein high-fat diet [35,36] (D12079B; Research Diets, New Brunswick, NJ 08901, USA) containing 40%, 42% and 17% kcal from fat (twice the requirement), carbohydrate and protein, respectively was modified to contain either 0.44 mg/kg Se and 2.4 mg/kg folate (high Se and folate; subsequently referred to as diet H; Table 1) or 0.08 mg/kg selenium and 0.4 mg/kg folate (low Se and folate; diet L). Analysis of the diets after manufacture confirmed that these were the actual levels of Se and folate. The levels of Se and folate in diet H are commonly used for supplementation in mouse and rat diets [37,38,39], and are approximately 3-fold and 5-fold higher, respectively, than the recommended requirements as described in the National Research Council (NRC) guidelines for Laboratory Animals [40]. For diet L, the levels of Se and folate were approximately 50% and 80% of the respective NRC guidelines. Additional ethoxyquin was included in both diets to prevent rancidity.

Thirty female wild type C57BL/6 mice (Animal Resource Centre, Western Australia) were fed either the high-fat diet supplemented with Se and folate (diet H; *n* = 15), or marginally deficient in Se and folate (diet L; *n* = 15) for 7 days prior to mating with male C57BL/6 mice (AgResearch Limited Ruakura Small Animal Facility, Hamilton, New Zealand) fed a standard rodent chow diet. Once pregnancy was confirmed via the presence of a vaginal plug, males were removed from the mating cages. The breeding dams consumed the diets during mating, gestation and lactation. Offspring remained with their dams until weaning (*c*. 28 days of age). At weaning, male offspring born to mothers fed diet H were randomly allocated to either continue on the same diet (HH; *n* = 6) or were switched to diet L (HL; *n* = 6) until 12 weeks of age; similarly, male offspring born to mothers fed diet L were randomly allocated to either continue on the same diet (LL; *n* = 6) or were switched to diet H (LH; *n* = 6). Mice were offered 20 g of food pellets twice weekly and had *ad libitum* access to water. Food intake (estimated by collecting and weighing uneaten food) and bodyweight were determined twice weekly. 

### 2.2. Sample Collection

Mice were euthanised at 12 weeks of age via CO_2_ asphyxiation and cervical dislocation. Prior to tissue collection, mice were fasted and re-fed to minimise variation in food intake immediately before sampling [41]. Colon tissue and liver were excised, washed in cold saline and stored in tube containing 1 mL of RNA*later*^®^ (Life Technologies NZ Ltd., Penrose, Auckland, New Zealand), then stored at 4 °C overnight. The RNA*later* was removed and the tissues snap-frozen in liquid nitrogen and stored at −85 °C until analysis.

**Table 1:** Composition of experimental diets (as formulated).

Macronutrient	Diet “L” (0.4 mg Folate + 0.08 mg Se/kg)		Diet “H” (2.4 mg Folate+ 0.44 mg Se/kg)		
	gm %	kcal%		gm %	kcal%
Protein	20	17		20	17
Carbohydrate	49	42		50	43
Fat	21	40		21	40
Total		99			100
kcal/g	4.68			4.68	
**Ingredient**	**gm**	**kcal**		**gm**	**kcal**
Casein	195	780		195	780
dl-methionine	3	12		3	12
Corn Starch	50	200		50	200
Matodextrin 10	100	400		100	400
Sucrose	341	1364		341	1364
Cellulose BW200	50	0		50	0
Milk Fat Anhydrous	200	1800		200	1800
Corn Oil	10	90		10	90
Mineral Mix S10001 †	0			35	
Sodium Selenite (45.7% Se)	0.000067			0.00058	
Mineral mix S19101 (no Se)	35			0	
Calcium Carbonate	4			4	
Vitamin Mix V10001 ‡	0			10	40
Vitamin mix V14901 (no Fo)	10	40		0	
Folic Acid	0.00032			0.00032	
Choline Bitartrate	2			2	
Cholesterol USP	1.5			1.5	
Ethoxyquin	0.04			0.04	
FD&C Yellow Dye #5	0.05			0	
FD&C Red Dye #40	0			0.05	
**Total**	**1001.59**	**4686**		**1001.59**	**4686**

† S1001: Calcium Phosphate, Dibasic, 29.5% Ca, 22.8% P 500 gm; Magnesium Oxide, 60.3% Mg 24 gm; Potassium Citrate, 1 H2O, 36.2% K 220 mg; Potassium Sulfate, 44.9% K, 18.4% S 52 gm; Sodium Chloride, 39.3% Na, 60.7% Cl 74 gm; Chromium K Sulfate, 12 H_2_O, 10.4% Cr 0.55 gm; Cupric Carbonate, 57.5% Cu 0.3 gm; Potassium Iodate, 59.3% I 0.01 gm; Ferric Citrate, 21.2% Fe 6 gm; Manganous Carbonate, 47.8% Mn 3.5 gm; Sodium Selenite, 45.7% Se 0.01 gm (not present in S19101); Zinc Carbonate, 52.1% Zn 1.6 gm; Sucrose 118.03 gm. ‡ V10001: Vitamin A Palmitate, 500,000 IU/gm 0.8 gm; Vitamin D3, 100,000 IU/gm 1 gm; Vitamin E Acetate, 500 IU/gm 10 gm; Menadione Sodium Bisulfite, 62.5% menadione 0.8 gm; Biotin, 1.0% 2 gm; Cyancocobalamin, 0.1% 1 gm; Folic Acid 0.2 gm; Nicotinic Acid 3 gm; Calcium Pantothenate 1.6 gm; Pyridoxine-HCl 0.7 gm; Riboflavin 0.6 gm; Thiamin HCl 0.6 gm; Sucrose 978.42 gm.

### 2.3. RNA and DNA Isolation

Genomic DNA, total RNA, and protein from whole colon and liver tissue were extracted using an AllPrep^®^ DNA/RNA/Protein mini kit (Qiagen, Hilden, Germany). Total RNA was quantified using a NanoDrop ND1000 and RNA quality was determined using an Agilent 2100 Bioanalyser (Agilent Technologies, Inc., Palo Alto, CA, USA) to measure the RNA integrity number (RIN); RNA with a RIN of 8.0 or higher was considered to be of sufficiently high quality for subsequent microarray analysis. RNA was stored at −85 °C until required.

### 2.4. Microarray Hybridisation and Analysis

Microarray hybridisation has been described in detail previously [42]. Briefly, a reference design was used for microarray hybridisation: intestinal and liver RNA extracts from all mice were pooled in an equimolar proportion and used as the reference sample. Cy3-labelled sample cRNA (0.75 μg) and Cy5-labelled reference cRNA (0.75 μg) was prepared using an *in situ* hybridisation kit-plus (Agilent Technologies, Inc.) and hybridised to Agilent Technologies Mouse G4122F—4 × 44 k 60 mer oligonucleotide arrays, as previously described [43]. Slides were scanned using an Agilent scanner after an automatic gain and calibration prior to each scan. Spot identification and quantification were performed using Agilent Feature Extraction software version 9.5. Microarray fluorescence signals were normalised using a global loess algorithm in R 3.0.2 with the Linear Models for Microarray Data (limma) package [44]. Differentially expressed genes were determined using an Empirical Bayes modified *t*-statistic from a linear model of microarray analysis. Probes with a greater than 1.5-fold change difference and FDR < 0.05 were considered differentially expressed. Over-representation analysis of KEGG and Reactome pathways and Gene Ontology processes was performed using the ClueGO application [45] in Cytoscape, with pathways and processes showing Bonferroni step down adjusted *p* < 0.01 considered significantly over-represented. Partial least squares-discriminant analysis (PLS-DA) was performed on genes that showed the highest variation across all samples (top 5% coefficient of variation) using the mixOmics package [46] in R. PLS-DA is a multivariate analysis that is commonly used for classification purposes by maximising the explained variance between groups. It is an appropriate choice for this particular study because a relationship between the multivariate gene expression data and the categorical variable, in our case the nutritional regime of dams and pups, is determined in such a way that the categorical variable values can by predicted for samples based on the gene expression profiles [47].

Gene expression data were also analysed by gene set enrichment analysis (GSEA) which tests for coordinated shifts in expression of whole sets of genes, rather than individual genes [48], because genes within biological pathways are not necessarily independent, which is assumed in per gene analyses. Furthermore, GSEA has been shown to be more sensitive than traditional per gene analyses [49,50]. GSEA was performed using the mroast function in the limma package, which estimates *p*-values by random rotations using Monte Carlo simulations. The gene sets used were the Reactome *Mus muscularis* pathways [51], with pathways showing *p* < 0.01 considered significantly different in expression.

The data discussed in this publication have been deposited in NCBI’s Gene Expression Omnibus [52] and are accessible through GEO Series accession number GSE68179.

## 3. Results

### 3.1. Food Intake and Body Weight

There was no effect of Se and folate supplementation/restriction either during gestation and weaning or post-weaning on the food intake or bodyweight of offspring at 28 days (*p* > 0.05).

### 3.2. Gene Expression in the Liver

**Figure 1:**
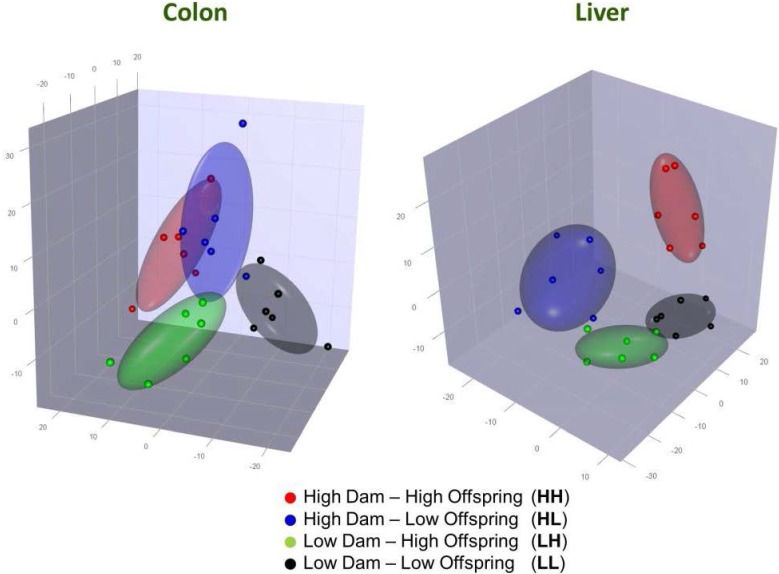
Partial least squares-discriminant analysis (PLS-DA) of gene expression profiles in colon (**left**) and liver (**right**) tissue for genes within the top 5% coefficient of variation; HH (red; *n* = 6), HL (blue; *n* = 6), LH (green; *n* = 6), LL (black; *n* = 6). Ellipses show 50% confidence interval boundary.

As can be seen in Figure 1, PLS-DA plots of the microarray data showed that overall gene expression patterns in the liver could be distinguished between offspring from each of the four treatment groups (HH, HL, LH, and LL). PLS-DA indicated that feeding diet L during gestation and lactation had the greatest effect on gene expression and this was also evident from the total number of genes found to show a significant change in expression with a fold change >|1.5| and FDR < 0.05 cut-off (Table 2); 720 probes showed a significant change when comparing HH *vs.* LH, 110 genes when comparing HH *vs.* HL, and 76 genes when comparing HH *vs.* LL. Comparison of other treatments showed smaller effects on gene expression and therefore further analysis focussed on the effects of altered Se/folate levels either during gestation and lactation (HH *vs.* LH), or during weaning (HH *vs.* HL). 

Over-representation analysis of differentially expressed genes assigned to the KEGG, Reactome, and Gene Ontology pathways and processes using the ClueGO application showed that gene expression changes in the liver when comparing HH and LH offspring included those involved in linoleic acid and retinol metabolism, hormone synthesis, Phase 1 metabolism, and DNA binding (Table S1). Processes and pathways significantly over-represented among differentially expressed genes in the liver of HH offspring compared with HL offspring were associated with a wide range of processes, the most significant of which were involved in lipid metabolism, mitochondrial metabolism, response to stress, and immune responses (Table S2).

Gene set enrichment analysis, which examines changes in expression of groups of genes, showed that feeding a Se/folate deficient diet during gestation and lactation, but not post-weaning, had major effects on a large number of pathways, including methyl metabolism pathways, translational control related pathways, mitochondrial metabolism, metal transporter and unfolded protein response pathways in the liver (HH *vs.* LH offspring; Table S3). This was reflected in the expression of *Rpl41*, *Rps19*, *Hsp90b1*, *Pfdn1* and *Xbp1* (all involved in translational control) as well as *Gpx4* and *Ndufb4* that have mitochondrial function (Table S4). 

Similarly, GSEA showed that expression of components of methyl metabolism and translational control pathways changed significantly in the liver when the Se/folate deficient diet was fed post-weaning (HH *vs.* HL) and, in addition, this treatment also showed significant changes in metal ion SLC transporter and zinc transporter pathways (Tables S5 and S6). Changes in lipid metabolism pathways were also identified by GSEA, which matches results from over-representation analysis of differentially expressed genes between offspring in the HH and HL groups. Interestingly, comparison of expression by GSEA in liver from animals supplemented with Se/folate either during gestation and lactation, or after weaning, with the animals fed the deficient diet throughout showed changes in translational control pathways (data not shown).

**Table 2:** Summary of gene expression changes in liver tissue in response to altered Se/folate levels. Comparisons for which further analyses, including GSEA, were carried out are marked *. For each of the experimental groups, data represent *n* = 6 animals. DE = differentially expressed.

Change in Gene Expression	Comparison				
	HH *vs.* HL*	LH *vs.* LL	HH *vs.* LL	HL *vs.* LL	HH *vs.* LH*
Lower expression	50	18	13	0	358
No change	43,269	43,356	43,303	43,379	42,659
Higher expression	60	5	63	0	362
Total DE genes	110	23	76	0	720

### 3.3. Gene Expression in the Colon

PLS-DA of the microarray data indicated that overall gene expression patterns in the colon could be distinguished between offspring from each of the four groups (HH, HL, LH, and LL; Figure 1). However, changes in gene expression were subtle as no genes were considered differentially expressed in the colon using criteria FDR < 0.05 and fold change >|1.5|. 

**Figure 2:**
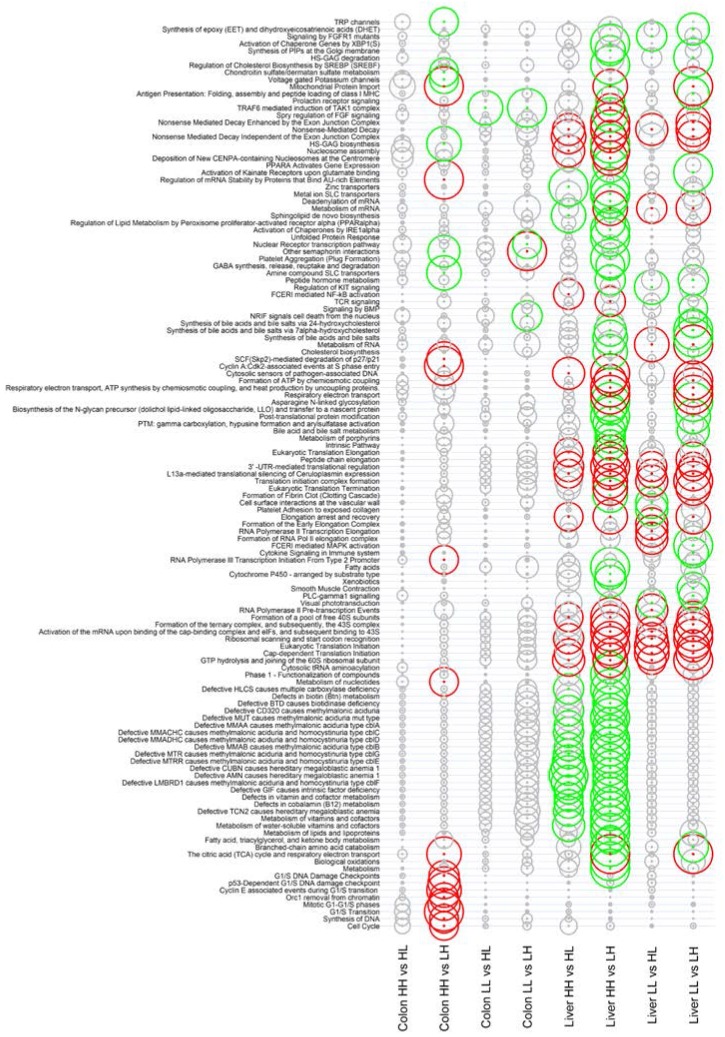
Gene set enrichment analysis (GSEA) of Reactome pathway expression. Size of the circles is proportional to significance (log odds), with increased circle diameter indicating increased significance. Pathways showing difference in expression with significance < 0.01 (*i.e.*, log odds > 2) are coloured red or green depending on the direction of expression shift. Red indicates significantly higher expression in first group, while green indicates higher expression in second group. GSEA of liver comparisons are shown in Tables S3 and S5, differentially expressed genes in the liver are listed in Tables S4 and S6, and GSEA of colon is shown in Table S7. Data represent comparisons between treatments with *n* = 6 animals in each treatment group.

Although no differentially expressed genes were seen in the colon, the greater sensitivity of GSEA resulted in identification of a number of differentially expressed pathways (*p* < 0.01). Gene set enrichment analysis showed that a deficiency in Se/folate during gestation and lactation (HH *vs.* LH) led to changes in cell cycle regulation pathways, DNA damage regulation, RNA processing, and the pathway of mitochondrial protein import (Figure 2; Table S7).

## 4. Discussion

In this study mice were fed a high-fat diet either supplemented with, or marginally deficient in, Se/folate to provide an experimental model of a human high-fat diet containing either sufficient or sub-optimal (rather than being severely deficient) levels of these two micronutrients. A cross-over design was used to investigate the effects of these diets fed to the mother during gestation and lactation compared with feeding to the offspring post-weaning. Microarray analysis of gene expression patterns following feeding of the two diets showed that there were different effects on gene expression in the liver and colon, and that the impact of the low Se/folate diet on the offspring is considerably greater when fed to the dams during gestation and lactation, rather than to the offspring themselves after weaning. Analysis of gene expression in the liver using GSEA showed that, although the magnitude of the effects was greater in response to feeding to the dams, feeding of the low Se/folate diet either during gestation/lactation or post-weaning had similar effects on pathways involved in regulation of translation (protein biosynthesis), methyl group metabolism, metal ion transporters and unfolded protein response. No significant differences were observed at the individual gene level in the colon between the different treatments. However, some differences in expression of Reactome pathways were seen in the colon, largely in pathways associated with cell cycle control; as was the case in liver, effects were greater if the deficient diet was fed to the dams during gestation and lactation, rather than to the offspring after weaning. 

Effects of the low Se/folate diet on methyl group metabolism pathways in the liver are not surprising because folate has a well-recognised role in S-adenosyl methionine metabolism and methylation reactions. In addition, the observed changes may be exacerbated by Se restriction because the feeding of low Se diets has previously been found to affect homocysteine levels and 1-carbon metabolism in the liver [25]. This is supported by a recent genome-wide association study, in which evidence of a genetic link between Se and homocysteine pathways is reported [53]. The effects on protein biosynthesis pathways may be related to the sub-optimal Se in the diet since in humans a modest Se supplementation has previously been observed to affect translational control/ protein biosynthesis pathways in lymphocytes [54]. This may also be related to sub-optimal dietary folate, which is necessary for the remethylation of homocysteine to methionine, with consequent implications for protein synthesis pathways. Alterations in translational signalling pathways are known to occur in response to a variety of stresses, including endoplasmic reticulum stress [55], and therefore the observed changes in such signalling pathways may reflect the fact that after feeding the low Se/folate diet the liver is under metabolic stress, and that this results in a stress-induced translational repression. In addition Se, as selenocysteine, is incorporated into selenoproteins during translation so it is likely that low Se supply leads to changes in the machinery regulating mRNA stability, turnover and translation [14,56]. It is not clear why these dietary changes cause altered expression of liver metal, SLC and zinc transporters but this could reflect compensatory changes in transporters to increase nutrient availability. 

Other pathways significantly affected by low Se/folate during gestation and lactation relate to the unfolded protein response and mitochondrial function; both of these may be a response to low Se because several selenoproteins are involved in the endoplasmic reticulum stress responses [14]. Furthermore, one selenoprotein (GPx4), the expression of which was found to be changed in this experiment, has been reported to have a role in mitochondrial function and knockdown of GPx4 affects expression of components of the respiratory chain [57]. GPx4 is regarded as being relatively high in the selenoprotein hierarchy and unaffected by Se depletion [56] but it is the only selenoprotein whose gene expression was sensitive to Se/folate restriction in the present work; this may reflect the importance of GPx4 in early life, as demonstrated by the gene knock-out being lethal [58].

In the liver, fatty acid/lipoprotein metabolism was the highest ranked pathway using CLUEGO and also found to be changed in the GSEA analysis. Such changes are compatible with earlier observations that in weanling rats or mice, low Se diets induce changes in fatty acid metabolism [25,59] and low folate in the maternal diet has been reported to affect the metabolism of the offspring and its response to a high-fat diet [1,24]. 

The pattern of gene expression in the colon responded differently to the low Se/folate diet compared with the liver. Again, changes were more evident if the deficient diet was fed to the dam during gestation and lactation (*i.e.*, the HH *vs.* LH comparison) rather than to the offspring post-weaning (HH *vs.* HL), but in this case changes were observed in mitochondrial protein import pathway and a number of pathways related to cell cycle regulation. Changes in the mitochondrial protein import pathway within the colon observed in this study may reflect altered metabolic control in response to a high fat diet, because this pathway is regarded as being closely involved in metabolic control [60]. The change in cell cycle regulation reflect changes in response to altered supply of both Se and folate because both have been implicated in processes relating to cell proliferation; this is likely to be important in a rapidly dividing cell population such as the colonic epithelium. 

## 5. Conclusions

In summary, the present transcriptomic analysis shows that feeding mice a high-fat diet marginally deficient in Se and folate during gestation and lactation has more effects on gene expression in the liver than the colon of the offspring and that, critically, feeding a low Se/folate maternal diet has dramatically more effects on the pattern of gene expression in the offspring than when this diet is fed to the offspring post-weaning. The latter result indicates that low Se and folate *in utero* and during lactation has metabolic effects that persist in the offspring in later life. In terms of translating these results to humans this lends further support to the importance of micronutrients in the maternal diet during pregnancy [3], and suggests that for mothers eating a high-fat diet, obtaining adequate Se and folate in the diet, and the combination of these two micronutrients, is important in determining metabolic parameters of their children in later life.

## 6. Supplementary Materials

Supplementary information is published online alongside the manuscript, consisting of seven supplementary tables as follows: Table S1: Over-representation analysis of genes differentially expressed in the liver of HH offspring compared with LH offspring generated using the ClueGO application; Table S2: Over-representation analysis of genes differentially expressed in the liver of HH offspring compared with HL offspring generated using the ClueGO application; Table S3: Gene set enrichment analysis for liver of HH offspring compared with LH offspring; Table S4: Genes differentially expressed in the liver of offspring in the HH group compared with those in the LH group. Table S5: Gene set enrichment analysis for liver of HH offspring compared with HL offspring; Table S6: Genes differentially expressed in the liver of offspring in the HH group compared with those in the HL group; Table S7: Gene set enrichment analysis for colon of HH offspring compared with LH offspring. All data represent comparisons between treatments with *n* = 6 animals in each treatment group.
