# Supplementary material for: Low Folate and Selenium in the Mouse Maternal Diet Alters Liver Gene Expression Patterns in the Offspring after Weaning
Source: Nutrients. 2015 May 8;7(5):3370–86. doi: 10.3390/nu7053370 (PMC4446756; doi:10.3390/nu7053370)
Supplement: Supplementary file 1 [file nutrients-07-03370-s001.docx]

**Supplementary Information**

**Table S1.** Over-representation analysis of genes differentially expressed in the liver of HH offspring compared with LH offspring generated using the ClueGO application.

| **GO ID/Pathway ID** | **GO Term/Pathway** | **Nr. Genes** | **% Associated Genes** | ***p* Value** | **Bonferroni *p* Value** |
| --- | --- | --- | --- | --- | --- |
| REACTOME:5416724 | Metabolism of lipids and lipoproteins | 47 | 7.730263 | 1.14E-18 | 7.52E-16 |
| REACTOME:5416688 | Metabolism | 80 | 4.761905 | 4.11E-18 | 2.71E-15 |
| GO:0044281 | small molecule metabolic process | 91 | 4.244403 | 2.10E-17 | 1.38E-14 |
| GO:0055114 | oxidation-reduction process | 52 | 5.485232 | 2.60E-14 | 1.71E-11 |
| GO:0071702 | organic substance transport | 78 | 4.094488 | 4.51E-14 | 2.96E-11 |
| GO:0006629 | lipid metabolic process | 54 | 5.202312 | 6.77E-14 | 4.44E-11 |
| GO:0048584 | positive regulation of response to stimulus | 62 | 4.704097 | 7.20E-14 | 4.72E-11 |
| GO:0006952 | defense response | 52 | 5.2 | 2.10E-13 | 1.37E-10 |
| GO:0070887 | cellular response to chemical stimulus | 70 | 4.181601 | 4.27E-13 | 2.79E-10 |
| GO:0006082 | organic acid metabolic process | 45 | 5.555555 | 1.07E-12 | 6.95E-10 |
| GO:0019752 | carboxylic acid metabolic process | 42 | 5.614973 | 4.54E-12 | 2.96E-09 |
| GO:0050790 | regulation of catalytic activity | 66 | 4.07911 | 6.67E-12 | 4.34E-09 |
| GO:0043436 | oxoacid metabolic process | 43 | 5.40201 | 8.76E-12 | 5.69E-09 |
| GO:0043085 | positive regulation of catalytic activity | 44 | 5.294826 | 9.55E-12 | 6.19E-09 |
| GO:0008202 | steroid metabolic process | 24 | 9.195402 | 1.14E-11 | 7.41E-09 |
| GO:1902531 | regulation of intracellular signal transduction | 57 | 4.401545 | 1.19E-11 | 7.66E-09 |
| GO:0044093 | positive regulation of molecular function | 50 | 4.725898 | 2.06E-11 | 1.33E-08 |
| GO:0060326 | cell chemotaxis | 20 | 11.04972 | 2.21E-11 | 1.42E-08 |
| GO:0080134 | regulation of response to stress | 41 | 5.359477 | 3.52E-11 | 2.26E-08 |
| GO:0009611 | response to wounding | 40 | 5.442177 | 3.91E-11 | 2.51E-08 |
| GO:0032787 | monocarboxylic acid metabolic process | 29 | 7.073171 | 5.23E-11 | 3.35E-08 |
| GO:0044255 | cellular lipid metabolic process | 39 | 5.394191 | 9.02E-11 | 5.78E-08 |
| GO:0008610 | lipid biosynthetic process | 31 | 6.444906 | 1.15E-10 | 7.37E-08 |
| GO:0044711 | single-organism biosynthetic process | 50 | 4.405286 | 2.41E-10 | 1.54E-07 |
| GO:0023056 | positive regulation of signaling | 46 | 4.505387 | 6.58E-10 | 4.19E-07 |
| GO:0010647 | positive regulation of cell communication | 46 | 4.487805 | 7.45E-10 | 4.74E-07 |

**Table S1.** *Cont.*

| **GO ID/Pathway ID** | **GO Term/Pathway** | **Nr. Genes** | **% Associated Genes** | ***p* Value** | **Bonferroni *p* Value** |
| --- | --- | --- | --- | --- | --- |
| GO:0033554 | cellular response to stress | 50 | 4.237288 | 9.02E-10 | 5.72E-07 |
| GO:0032101 | regulation of response to external stimulus | 30 | 6.048387 | 1.05E-09 | 6.65E-07 |
| GO:0006954 | inflammatory response | 29 | 6.170213 | 1.27E-09 | 8.06E-07 |
| GO:0009967 | positive regulation of signal transduction | 43 | 4.479167 | 2.95E-09 | 1.86E-06 |
| KEGG:04141 | Protein processing in endoplasmic reticulum | 17 | 10.05917 | 3.05E-09 | 1.92E-06 |
| GO:0031399 | regulation of protein modification process | 45 | 4.326923 | 3.56E-09 | 2.25E-06 |
| GO:0006066 | alcohol metabolic process | 21 | 7.749077 | 5.05E-09 | 3.17E-06 |
| GO:1901615 | organic hydroxy compound metabolic process | 25 | 6.56168 | 5.29E-09 | 3.32E-06 |
| GO:0042592 | homeostatic process | 49 | 4.059652 | 5.43E-09 | 3.40E-06 |
| GO:1902533 | positive regulation of intracellular signal transduction | 33 | 5.229794 | 5.77E-09 | 3.61E-06 |
| GO:0042981 | regulation of apoptotic process | 48 | 4.071247 | 7.21E-09 | 4.51E-06 |
| GO:0001775 | cell activation | 35 | 4.895105 | 1.06E-08 | 6.60E-06 |
| GO:0043067 | regulation of programmed cell death | 48 | 4.013378 | 1.13E-08 | 7.04E-06 |
| GO:0032270 | positive regulation of cellular protein metabolic process | 37 | 4.701398 | 1.14E-08 | 7.10E-06 |
| GO:0044242 | cellular lipid catabolic process | 15 | 10.56338 | 1.33E-08 | 8.29E-06 |
| GO:0031401 | positive regulation of protein modification process | 34 | 4.934688 | 1.42E-08 | 8.82E-06 |
| GO:0006694 | steroid biosynthetic process | 15 | 10.34483 | 1.78E-08 | 1.10E-05 |
| GO:0051247 | positive regulation of protein metabolic process | 39 | 4.467354 | 1.80E-08 | 1.11E-05 |
| GO:0016482 | cytoplasmic transport | 32 | 5.095542 | 1.82E-08 | 1.13E-05 |
| GO:0032103 | positive regulation of response to external stimulus | 17 | 8.854167 | 2.10E-08 | 1.29E-05 |
| GO:0001932 | regulation of protein phosphorylation | 38 | 4.497041 | 2.33E-08 | 1.43E-05 |
| GO:0006641 | triglyceride metabolic process | 11 | 15.27778 | 2.49E-08 | 1.53E-05 |
| GO:0045859 | regulation of protein kinase activity | 29 | 5.360444 | 2.92E-08 | 1.79E-05 |
| GO:0048585 | negative regulation of response to stimulus | 41 | 4.222451 | 3.64E-08 | 2.23E-05 |
| GO:0006631 | fatty acid metabolic process | 21 | 6.885246 | 3.97E-08 | 2.43E-05 |
| GO:0006935 | chemotaxis | 25 | 5.924171 | 3.99E-08 | 2.43E-05 |
| GO:0042330 | taxis | 25 | 5.910165 | 4.18E-08 | 2.54E-05 |
| GO:0048870 | cell motility | 42 | 4.137931 | 4.31E-08 | 2.62E-05 |

**Table S1.** *Cont.*

| **GO ID/Pathway ID** | **GO Term/Pathway** | **Nr. Genes** | **% Associated Genes** | ***p* Value** | **Bonferroni *p* Value** |
| --- | --- | --- | --- | --- | --- |
| GO:0016125 | sterol metabolic process | 13 | 11.50443 | 4.48E-08 | 2.72E-05 |
| GO:0044283 | small molecule biosynthetic process | 24 | 6.075949 | 4.66E-08 | 2.82E-05 |
| REACTOME:5416723 | Fatty acid, triacylglycerol, and ketone body metabolism | 17 | 8.333333 | 5.13E-08 | 3.11E-05 |
| GO:0072594 | establishment of protein localization to organelle | 21 | 6.774194 | 5.24E-08 | 3.17E-05 |
| GO:0010562 | positive regulation of phosphorus metabolic process | 35 | 4.557292 | 6.20E-08 | 3.74E-05 |
| GO:0045937 | positive regulation of phosphate metabolic process | 35 | 4.557292 | 6.20E-08 | 3.74E-05 |
| GO:0050870 | positive regulation of T cell activation | 14 | 10.14493 | 6.81E-08 | 4.10E-05 |
| REACTOME:5417730 | PPARA Activates Gene Expression | 14 | 10.07194 | 7.46E-08 | 4.48E-05 |
| GO:0048247 | lymphocyte chemotaxis | 7 | 29.16667 | 8.24E-08 | 4.94E-05 |
| REACTOME:5417626 | Regulation of Lipid Metabolism by Peroxisome proliferator-activated receptor alpha (PPARalpha) | 14 | 9.929078 | 8.93E-08 | 5.35E-05 |
| GO:0016042 | lipid catabolic process | 18 | 7.563025 | 9.17E-08 | 5.49E-05 |
| GO:0045860 | positive regulation of protein kinase activity | 22 | 6.285714 | 9.44E-08 | 5.64E-05 |
| GO:0016477 | cell migration | 39 | 4.193548 | 9.54E-08 | 5.69E-05 |
| GO:0002696 | positive regulation of leukocyte activation | 17 | 7.834101 | 1.26E-07 | 7.50E-05 |
| GO:0006639 | acylglycerol metabolic process | 11 | 13.09524 | 1.28E-07 | 7.62E-05 |
| GO:0042327 | positive regulation of phosphorylation | 32 | 4.671533 | 1.34E-07 | 7.93E-05 |
| GO:0043549 | regulation of kinase activity | 29 | 4.991394 | 1.34E-07 | 7.93E-05 |
| REACTOME:5416919 | Cytochrome P450—arranged by substrate type | 11 | 12.94118 | 1.45E-07 | 8.58E-05 |
| GO:0051251 | positive regulation of lymphocyte activation | 16 | 8.205129 | 1.57E-07 | 9.27E-05 |
| GO:0023014 | signal transduction by phosphorylation | 29 | 4.948805 | 1.60E-07 | 9.43E-05 |
| GO:0006638 | neutral lipid metabolic process | 11 | 12.7907 | 1.64E-07 | 9.64E-05 |
| GO:0006869 | lipid transport | 17 | 7.657658 | 1.75E-07 | 1.03E-04 |
| GO:0008203 | cholesterol metabolic process | 12 | 11.21495 | 1.97E-07 | 1.15E-04 |
| GO:0051272 | positive regulation of cellular component movement | 20 | 6.514658 | 2.05E-07 | 1.20E-04 |
| GO:0000165 | MAPK cascade | 28 | 4.973357 | 2.38E-07 | 1.39E-04 |
| GO:0050867 | positive regulation of cell activation | 17 | 7.488987 | 2.40E-07 | 1.40E-04 |

**Table S1.** *Cont.*

| **GO ID/Pathway ID** | **GO Term/Pathway** | **Nr. Genes** | **% Associated Genes** | ***p* Value** | **Bonferroni *p* Value** |
| --- | --- | --- | --- | --- | --- |
| GO:0051336 | regulation of hydrolase activity | 38 | 4.099245 | 2.49E-07 | 1.45E-04 |
| GO:0006605 | protein targeting | 22 | 5.929919 | 2.58E-07 | 1.50E-04 |
| GO:0033674 | positive regulation of kinase activity | 22 | 5.913979 | 2.70E-07 | 1.57E-04 |
| GO:0001934 | positive regulation of protein phosphorylation | 28 | 4.903678 | 3.16E-07 | 1.83E-04 |
| GO:0051338 | regulation of transferase activity | 29 | 4.769737 | 3.44E-07 | 1.99E-04 |
| GO:0031347 | regulation of defense response | 22 | 5.789474 | 3.88E-07 | 2.24E-04 |
| GO:0033365 | protein localization to organelle | 24 | 5.405406 | 4.00E-07 | 2.31E-04 |
| GO:0010648 | negative regulation of cell communication | 35 | 4.191617 | 4.52E-07 | 2.60E-04 |
| GO:0001816 | cytokine production | 25 | 5.208334 | 4.57E-07 | 2.62E-04 |
| REACTOME:5417363 | Mineralocorticoid biosynthesis | 5 | 45.45454 | 4.93E-07 | 2.82E-04 |
| GO:0051347 | positive regulation of transferase activity | 22 | 5.699482 | 5.06E-07 | 2.89E-04 |
| GO:0016053 | organic acid biosynthetic process | 18 | 6.69145 | 5.65E-07 | 3.23E-04 |
| GO:0046394 | carboxylic acid biosynthetic process | 18 | 6.69145 | 5.65E-07 | 3.23E-04 |
| GO:0050729 | positive regulation of inflammatory response | 10 | 12.82051 | 5.88E-07 | 3.35E-04 |
| GO:0017038 | protein import | 17 | 6.854839 | 8.34E-07 | 4.75E-04 |
| REACTOME:5416762 | Phase 1—Functionalization of compounds | 11 | 10.89109 | 8.55E-07 | 4.86E-04 |
| GO:0072329 | monocarboxylic acid catabolic process | 10 | 12.19512 | 9.43E-07 | 5.34E-04 |
| GO:0015711 | organic anion transport | 19 | 6.148867 | 9.90E-07 | 5.60E-04 |
| GO:0043408 | regulation of MAPK cascade | 25 | 4.99002 | 9.99E-07 | 5.64E-04 |
| GO:0009968 | negative regulation of signal transduction | 33 | 4.182509 | 1.02E-06 | 5.77E-04 |
| GO:0046890 | regulation of lipid biosynthetic process | 12 | 9.523809 | 1.17E-06 | 6.57E-04 |
| GO:0010876 | lipid localization | 17 | 6.666667 | 1.23E-06 | 6.89E-04 |
| GO:0050778 | positive regulation of immune response | 20 | 5.813953 | 1.23E-06 | 6.90E-04 |
| GO:0023057 | negative regulation of signaling | 34 | 4.076739 | 1.24E-06 | 6.92E-04 |
| REACTOME:5417369 | Glucocorticoid biosynthesis | 5 | 38.46154 | 1.34E-06 | 7.47E-04 |
| GO:0010035 | response to inorganic substance | 19 | 6.012658 | 1.38E-06 | 7.71E-04 |
| GO:0040017 | positive regulation of locomotion | 19 | 6.012658 | 1.38E-06 | 7.71E-04 |

**Table S1.** *Cont.*

| **GO ID/Pathway ID** | **GO Term/Pathway** | **Nr. Genes** | **% Associated Genes** | ***p* Value** | **Bonferroni *p* Value** |
| --- | --- | --- | --- | --- | --- |
| GO:0009894 | regulation of catabolic process | 31 | 4.269972 | 1.43E-06 | 7.96E-04 |
| GO:0031349 | positive regulation of defense response | 15 | 7.389163 | 1.45E-06 | 8.07E-04 |
| KEGG:00140 | Steroid hormone biosynthesis | 10 | 11.62791 | 1.47E-06 | 8.17E-04 |
| GO:0044092 | negative regulation of molecular function | 33 | 4.07911 | 1.76E-06 | 9.75E-04 |
| GO:0001817 | regulation of cytokine production | 22 | 5.275779 | 1.82E-06 | 0.001007 |
| GO:0030335 | positive regulation of cell migration | 18 | 6.164383 | 1.84E-06 | 0.001016 |
| GO:0009062 | fatty acid catabolic process | 9 | 13.04348 | 1.87E-06 | 0.001029 |
| GO:0043086 | negative regulation of catalytic activity | 28 | 4.46571 | 1.99E-06 | 0.001095 |
| REACTOME:5417506 | Platelet degranulation | 10 | 11.23596 | 2.02E-06 | 0.001111 |
| REACTOME:5417368 | Androgen biosynthesis | 5 | 35.71429 | 2.05E-06 | 0.001123 |
| GO:2000379 | positive regulation of reactive oxygen species metabolic process | 7 | 18.42105 | 2.46E-06 | 0.001348 |
| GO:2000147 | positive regulation of cell motility | 18 | 6.020067 | 2.57E-06 | 0.001404 |
| GO:0050863 | regulation of T cell activation | 15 | 7.042254 | 2.64E-06 | 0.00144 |
| KEGG:04976 | Bile secretion | 9 | 12.5 | 2.68E-06 | 0.00146 |
| GO:0006909 | phagocytosis | 11 | 9.649123 | 2.86E-06 | 0.001554 |
| GO:0046486 | glycerolipid metabolic process | 16 | 6.584362 | 2.95E-06 | 0.001601 |
| GO:0034976 | response to endoplasmic reticulum stress | 10 | 10.6383 | 3.35E-06 | 0.00181 |
| REACTOME:5417074 | Response to elevated platelet cytosolic Ca2+ | 10 | 10.6383 | 3.35E-06 | 0.00181 |
| GO:0007159 | leukocyte cell-cell adhesion | 7 | 17.5 | 3.54E-06 | 0.001911 |
| GO:0072593 | reactive oxygen species metabolic process | 12 | 8.571428 | 3.56E-06 | 0.001916 |
| GO:0006606 | protein import into nucleus | 15 | 6.818182 | 3.93E-06 | 0.002115 |
| GO:0044744 | protein targeting to nucleus | 15 | 6.818182 | 3.93E-06 | 0.002115 |
| GO:0050727 | regulation of inflammatory response | 15 | 6.818182 | 3.93E-06 | 0.002115 |
| GO:1902593 | single-organism nuclear import | 15 | 6.818182 | 3.93E-06 | 0.002115 |
| REACTOME:5416918 | Xenobiotics | 7 | 17.07317 | 4.21E-06 | 0.002256 |
| GO:0019725 | cellular homeostasis | 26 | 4.490501 | 4.20E-06 | 0.002256 |
| GO:0032763 | regulation of mast cell cytokine production | 3 | 100 | 4.38E-06 | 0.002341 |

**Table S1.** *Cont.*

| **GO ID/Pathway ID** | **GO Term/Pathway** | **Nr. Genes** | **% Associated Genes** | ***p* Value** | **Bonferroni *p* Value** |
| --- | --- | --- | --- | --- | --- |
| GO:0051170 | nuclear import | 15 | 6.756757 | 4.39E-06 | 0.002344 |
| GO:0045454 | cell redox homeostasis | 8 | 13.7931 | 4.57E-06 | 0.002436 |
| GO:0071900 | regulation of protein serine/threonine kinase activity | 19 | 5.507247 | 4.98E-06 | 0.00265 |
| GO:0052547 | regulation of peptidase activity | 20 | 5.291005 | 5.13E-06 | 0.002724 |
| GO:0042304 | regulation of fatty acid biosynthetic process | 6 | 21.42857 | 5.20E-06 | 0.002756 |
| GO:0001525 | angiogenesis | 20 | 5.249344 | 5.77E-06 | 0.003052 |
| GO:0050865 | regulation of cell activation | 20 | 5.235602 | 6.00E-06 | 0.003166 |
| REACTOME:5417072 | Platelet activation, signaling and aggregation | 14 | 7 | 6.08E-06 | 0.003206 |
| GO:0097190 | apoptotic signaling pathway | 24 | 4.597701 | 6.65E-06 | 0.003499 |
| GO:0010038 | response to metal ion | 13 | 7.428571 | 6.85E-06 | 0.003599 |
| KEGG:03320 | PPAR signaling pathway | 9 | 11.11111 | 7.23E-06 | 0.003787 |
| GO:0006820 | anion transport | 21 | 4.988123 | 7.41E-06 | 0.003873 |
| REACTOME:5417364 | Metabolism of steroid hormones and vitamin D | 6 | 20 | 7.97E-06 | 0.004161 |
| GO:0061082 | myeloid leukocyte cytokine production | 5 | 27.77778 | 8.31E-06 | 0.004329 |
| GO:0046470 | phosphatidylcholine metabolic process | 6 | 19.35484 | 9.75E-06 | 0.005069 |
| REACTOME:5417952 | Synthesis of (16–20)-hydroxyeicosatetraenoic acids (HETE) | 6 | 19.35484 | 9.75E-06 | 0.005069 |
| GO:0006886 | intracellular protein transport | 26 | 4.276316 | 9.96E-06 | 0.005168 |
| GO:0006913 | nucleocytoplasmic transport | 18 | 5.454546 | 1.00E-05 | 0.005205 |
| WP:1 | Statin Pathway | 5 | 26.31579 | 1.11E-05 | 0.005751 |
| GO:0006662 | glycerol ether metabolic process | 5 | 26.31579 | 1.11E-05 | 0.005751 |
| GO:0018904 | ether metabolic process | 5 | 26.31579 | 1.11E-05 | 0.005751 |
| GO:0060548 | negative regulation of cell death | 28 | 4.069768 | 1.13E-05 | 0.005843 |
| GO:0043410 | positive regulation of MAPK cascade | 18 | 5.389222 | 1.18E-05 | 0.006091 |
| GO:0051169 | nuclear transport | 18 | 5.389222 | 1.18E-05 | 0.006091 |
| GO:0019216 | regulation of lipid metabolic process | 15 | 6.17284 | 1.30E-05 | 0.006692 |
| GO:0048514 | blood vessel morphogenesis | 22 | 4.64135 | 1.39E-05 | 0.007137 |

**Table S1.** *Cont.*

| **GO ID/Pathway ID** | **GO Term/Pathway** | **Nr. Genes** | **% Associated Genes** | ***p* Value** | **Bonferroni *p* Value** |
| --- | --- | --- | --- | --- | --- |
| REACTOME:5417096 | Formation of Fibrin Clot (Clotting Cascade) | 6 | 18.18182 | 1.43E-05 | 0.007302 |
| KEGG:00830 | Retinol metabolism | 9 | 10.22727 | 1.43E-05 | 0.007311 |
| REACTOME:5417790 | Retinoid metabolism and transport | 7 | 14.28571 | 1.44E-05 | 0.007326 |
| GO:2000403 | positive regulation of lymphocyte migration | 5 | 25 | 1.46E-05 | 0.007448 |
| GO:0050671 | positive regulation of lymphocyte proliferation | 10 | 9.009009 | 1.49E-05 | 0.007568 |
| GO:0043066 | negative regulation of apoptotic process | 26 | 4.166667 | 1.56E-05 | 0.007912 |
| GO:0032946 | positive regulation of mononuclear cell proliferation | 10 | 8.928572 | 1.61E-05 | 0.008158 |
| GO:0044282 | small molecule catabolic process | 14 | 6.422019 | 1.62E-05 | 0.008183 |
| GO:0042439 | ethanolamine-containing compound metabolic process | 7 | 14 | 1.65E-05 | 0.0083 |
| GO:0042110 | T cell activation | 19 | 5.053192 | 1.68E-05 | 0.008439 |
| REACTOME:5417147 | Bile acid and bile salt metabolism | 6 | 17.64706 | 1.71E-05 | 0.008574 |
| GO:0032762 | mast cell cytokine production | 3 | 75 | 1.73E-05 | 0.008661 |
| GO:0002253 | activation of immune response | 15 | 6 | 1.82E-05 | 0.009099 |
| REACTOME:5417729 | BMAL1:CLOCK/NPAS2 Activates Circadian Expression | 5 | 23.80953 | 1.89E-05 | 0.009455 |

**Table S2.** Over-representation analysis of genes differentially expressed in the liver of HH offspring compared with HL offspring generated using the ClueGO application.

| **GO ID/Pathway ID** | **GO Term/Pathway** | **Nr. Genes** | **% Associated Genes** | ***p* Value** | **Bonferroni *p* Value** |
| --- | --- | --- | --- | --- | --- |
| REACTOME:5416918 | Xenobiotics | 5 | 12.19512 | 9.34E-08 | 2.15E-06 |
| GO:0020037 | heme binding | 6 | 4.651163 | 1.50E-06 | 3.30E-05 |
| GO:0046906 | tetrapyrrole binding | 6 | 4.379562 | 2.13E-06 | 4.47E-05 |
| REACTOME:5417870 | Synthesis of PE | 3 | 27.27273 | 3.21E-06 | 6.42E-05 |
| REACTOME:5416919 | Cytochrome P450—arranged by substrate type | 5 | 5.882353 | 3.72E-06 | 7.08E-05 |
| KEGG:00830 | Retinol metabolism | 5 | 5.681818 | 4.42E-06 | 7.95E-05 |
| REACTOME:5416762 | Phase 1—Functionalization of compounds | 5 | 4.950495 | 8.69E-06 | 1.48E-04 |
| GO:0001046 | core promoter sequence-specific DNA binding | 4 | 8.163265 | 1.00E-05 | 1.60E-04 |

**Table S2.** *Cont.*

| **GO ID/Pathway ID** | **GO Term/Pathway** | **Nr. Genes** | **% Associated Genes** | ***p* Value** | **Bonferroni *p* Value** |
| --- | --- | --- | --- | --- | --- |
| REACTOME:5417495 | Synthesis of PC | 3 | 16.66667 | 1.57E-05 | 2.35E-04 |
| WP:544 | Circadian Exercise | 4 | 6.557377 | 2.41E-05 | 3.37E-04 |
| REACTOME:5417953 | Synthesis of epoxy (EET) and dihydroxyeicosatrienoic acids (DHET) | 3 | 13.63636 | 2.93E-05 | 3.81E-04 |
| GO:0016712 | oxidoreductase activity, acting on paired donors, with incorporation or  reduction of molecular oxygen, reduced flavin or flavoprotein as one donor,  and incorporation of one atom of oxygen | 3 | 8.823529 | 1.11E-04 | 7.79E-04 |
| GO:0001077 | RNA polymerase II core promoter proximal region sequence-specific  DNA binding transcription factor activity involved in positive  regulation of transcription | 4 | 4.545455 | 1.02E-04 | 8.15E-04 |
| KEGG:00140 | Steroid hormone biosynthesis | 4 | 4.651163 | 9.31E-05 | 8.38E-04 |
| GO:0001047 | core promoter binding | 4 | 4.938272 | 7.37E-05 | 8.85E-04 |
| REACTOME:5417952 | Synthesis of (16–20)-hydroxyeicosatetraenoic acids (HETE) | 3 | 9.67742 | 8.41E-05 | 9.25E-04 |
| GO:0070330 | aromatase activity | 3 | 9.375 | 9.26E-05 | 9.26E-04 |
| GO:0048641 | regulation of skeletal muscle tissue development | 3 | 4.166667 | 0.001029 | 0.001029 |
| GO:0043401 | steroid hormone mediated signaling pathway | 3 | 5.084746 | 5.76E-04 | 0.001727 |
| GO:0071248 | cellular response to metal ion | 3 | 4.411765 | 8.72E-04 | 0.001744 |
| WP:1268 | Diurnally regulated genes with circadian orthologs | 3 | 5.454546 | 4.68E-04 | 0.001873 |
| GO:0003707 | steroid hormone receptor activity | 3 | 5.454546 | 4.68E-04 | 0.001873 |
| REACTOME:5417551 | Nuclear Receptor transcription pathway | 3 | 5.660378 | 4.20E-04 | 0.002099 |
| KEGG:00591 | Linoleic acid metabolism | 3 | 6 | 3.53E-04 | 0.002119 |

**Table S3.** Gene set enrichment analysis for liver of HH offspring compared with LH offspring.

| **PathID** | **Pathway** | **NGenes** | **PropDown** | **PropUp** | **Direction** | ***p* Value** |
| --- | --- | --- | --- | --- | --- | --- |
| 4809949 | Eukaryotic Translation Initiation | 182 | 0.120879 | 0.406593 | Up | 0.001998 |
| 4809875 | The citric acid (TCA) cycle and respiratory electron transport | 177 | 0.129944 | 0.378531 | Up | 0.001998 |
| 4810291 | 3' -UTR-mediated translational regulation | 166 | 0.108434 | 0.439759 | Up | 0.001998 |
| 4809956 | Formation of a pool of free 40S subunits | 141 | 0.06383 | 0.489362 | Up | 0.001998 |
| 4810336 | Asparagine N-linked glycosylation | 141 | 0.35461 | 0.056738 | Down | 0.001998 |
| 4810347 | Respiratory electron transport, ATP synthesis by chemiosmotic coupling,  and heat production by uncoupling proteins. | 116 | 0.112069 | 0.517241 | Up | 0.001998 |
| 4809953 | Activation of the mRNA upon binding of the cap-binding complex and eIFs,  and subsequent binding to 43S | 107 | 0.158879 | 0.383178 | Up | 0.001998 |
| 4810736 | Unfolded Protein Response | 106 | 0.367925 | 0.084906 | Down | 0.001998 |
| 4810346 | Respiratory electron transport | 89 | 0.101124 | 0.550562 | Up | 0.001998 |
| 4810738 | Activation of Chaperones by IRE1alpha | 83 | 0.373494 | 0.048193 | Down | 0.001998 |
| 4811060 | Regulation of Cholesterol Biosynthesis by SREBP (SREBF) | 73 | 0.465753 | 0.082192 | Down | 0.001998 |
| 4810902 | Nucleosome assembly | 55 | 0.090909 | 0.4 | Up | 0.001998 |
| 4810859 | Metal ion SLC transporters | 40 | 0.375 | 0.1 | Down | 0.001998 |
| 4810861 | Zinc transporters | 27 | 0.407407 | 0.037037 | Down | 0.001998 |
| 4810940 | Nonsense-Mediated Decay | 169 | 0.076923 | 0.420118 | Up | 0.001998 |
| 4810941 | Nonsense Mediated Decay Enhanced by the Exon Junction Complex | 169 | 0.076923 | 0.420118 | Up | 0.001998 |
| 4809954 | Formation of the ternary complex, and subsequently, the 43S complex | 90 | 0.1 | 0.422222 | Up | 0.001998 |
| 4811069 | Activation of Chaperone Genes by XBP1(S) | 82 | 0.378049 | 0.04878 | Down | 0.001998 |
| 4809909 | Defective MMADHC causes methylmalonic aciduria and homocystinuria type cblD | 123 | 0.317073 | 0.097561 | Down | 0.001998 |
| 4810686 | Platelet Aggregation (Plug Formation) | 73 | 0.246575 | 0.109589 | Down | 0.001998 |
| 4809931 | Phase 1—Functionalization of compounds | 126 | 0.420635 | 0.111111 | Down | 0.001998 |
| 4809900 | Defects in vitamin and cofactor metabolism | 123 | 0.317073 | 0.097561 | Down | 0.001998 |
| 4809908 | Defective MMAB causes methylmalonic aciduria type cblB | 123 | 0.317073 | 0.097561 | Down | 0.001998 |
| 4809887 | Branched-chain amino acid catabolism | 30 | 0.5 | 0.033333 | Down | 0.001998 |
| 4809912 | Defective MUT causes methylmalonic aciduria mut type | 123 | 0.317073 | 0.097561 | Down | 0.001998 |
| 4809896 | Metabolism of water-soluble vitamins and cofactors | 123 | 0.317073 | 0.097561 | Down | 0.001998 |

**Table S3.** *Cont.*

| **PathID** | **Pathway** | **NGenes** | **PropDown** | **PropUp** | **Direction** | **p Value** |
| --- | --- | --- | --- | --- | --- | --- |
| 4809897 | Metabolism of vitamins and cofactors | 123 | 0.317073 | 0.097561 | Down | 0.001998 |
| 4809898 | Defective TCN2 causes hereditary megaloblastic anemia | 123 | 0.317073 | 0.097561 | Down | 0.001998 |
| 4809907 | Defective MTR causes methylmalonic aciduria and homocystinuria type cblG | 123 | 0.317073 | 0.097561 | Down | 0.001998 |
| 4809916 | Defective HLCS causes multiple carboxylase deficiency | 123 | 0.317073 | 0.097561 | Down | 0.001998 |
| 4809863 | Biological oxidations | 216 | 0.347222 | 0.101852 | Down | 0.001998 |
| 4810092 | Fatty acids | 22 | 0.681818 | 0 | Down | 0.001998 |
| 4809894 | Metabolism of lipids and lipoproteins | 843 | 0.285884 | 0.115065 | Down | 0.001998 |
| 4810390 | Cytosolic sensors of pathogen-associated DNA | 92 | 0.130435 | 0.206522 | Up | 0.001998 |
| 4810325 | Post-translational protein modification | 286 | 0.262238 | 0.097902 | Down | 0.001998 |
| 4810912 | HS-GAG biosynthesis | 43 | 0.232558 | 0.046512 | Down | 0.001998 |
| 4809858 | Metabolism | 2292 | 0.232984 | 0.136562 | Down | 0.001998 |
| 4809947 | GTP hydrolysis and joining of the 60S ribosomal subunit | 172 | 0.116279 | 0.424419 | Up | 0.003996 |
| 4810293 | Eukaryotic Translation Elongation | 130 | 0.053846 | 0.523077 | Up | 0.003996 |
| 4810283 | Eukaryotic Translation Termination | 123 | 0.03252 | 0.536585 | Up | 0.003996 |
| 4810292 | Peptide chain elongation | 123 | 0.056911 | 0.536585 | Up | 0.003996 |
| 4809952 | Ribosomal scanning and start codon recognition | 106 | 0.150943 | 0.386792 | Up | 0.003996 |
| 4810899 | PPARA Activates Gene Expression | 98 | 0.459184 | 0.112245 | Down | 0.003996 |
| 4810221 | Elongation arrest and recovery | 50 | 0.12 | 0.34 | Up | 0.003996 |
| 4810289 | Translation initiation complex formation | 105 | 0.152381 | 0.390476 | Up | 0.003996 |
| 4810844 | Metabolism of mRNA | 371 | 0.153639 | 0.250674 | Up | 0.003996 |
| 4809913 | Defective CD320 causes methylmalonic aciduria | 123 | 0.317073 | 0.097561 | Down | 0.003996 |
| 4810520 | Cholesterol biosynthesis | 44 | 0.590909 | 0.022727 | Down | 0.003996 |
| 4810266 | Formation of Fibrin Clot (Clotting Cascade) | 41 | 0.439024 | 0.04878 | Down | 0.003996 |
| 4810324 | PTM: gamma carboxylation, hypusine formation and arylsulfatase activation | 32 | 0.375 | 0.09375 | Down | 0.003996 |
| 4809914 | Defective BTD causes biotidinase deficiency | 123 | 0.317073 | 0.097561 | Down | 0.003996 |
| 4810901 | Deposition of New CENPA-containing Nucleosomes at the Centromere | 55 | 0.090909 | 0.4 | Up | 0.003996 |

**Table S3.** *Cont.*

| **PathID** | **Pathway** | **NGenes** | **PropDown** | **PropUp** | **Direction** | **p Value** |
| --- | --- | --- | --- | --- | --- | --- |
| 4809899 | Defects in cobalamin (B12) metabolism | 123 | 0.317073 | 0.097561 | Down | 0.003996 |
| 4809910 | Defective MMACHC causes methylmalonic aciduria and homocystinuria type cblC | 123 | 0.317073 | 0.097561 | Down | 0.003996 |
| 4809915 | Defects in biotin (Btn) metabolism | 123 | 0.317073 | 0.097561 | Down | 0.003996 |
| 4810996 | Mitochondrial Protein Import | 73 | 0.123288 | 0.342466 | Up | 0.003996 |
| 4809904 | Defective AMN causes hereditary megaloblastic anemia 1 | 123 | 0.317073 | 0.097561 | Down | 0.003996 |
| 4809905 | Defective CUBN causes hereditary megaloblastic anemia 1 | 123 | 0.317073 | 0.097561 | Down | 0.003996 |
| 4809902 | Defective GIF causes intrinsic factor deficiency | 123 | 0.317073 | 0.097561 | Down | 0.003996 |
| 4809906 | Defective MTRR causes methylmalonic aciduria and homocystinuria type cblE | 123 | 0.317073 | 0.097561 | Down | 0.003996 |
| 4809911 | Defective MMAA causes methylmalonic aciduria type cblA | 123 | 0.317073 | 0.097561 | Down | 0.003996 |
| 4809903 | Defective LMBRD1 causes methylmalonic aciduria and homocystinuria type cblF | 123 | 0.317073 | 0.097561 | Down | 0.003996 |
| 4810845 | Deadenylation of mRNA | 55 | 0.327273 | 0.090909 | Down | 0.003996 |
| 4810526 | Synthesis of bile acids and bile salts | 34 | 0.411765 | 0.029412 | Down | 0.003996 |
| 4810943 | Spry regulation of FGF signaling | 27 | 0.259259 | 0.111111 | Down | 0.003996 |
| 4810939 | Nonsense Mediated Decay Independent of the Exon Junction Complex | 131 | 0.053435 | 0.519084 | Up | 0.005994 |
| 4810363 | Formation of ATP by chemiosmotic coupling | 22 | 0.181818 | 0.454545 | Up | 0.005994 |
| 4810290 | L13a-mediated translational silencing of Ceruloplasmin expression | 166 | 0.108434 | 0.439759 | Up | 0.005994 |
| 4810088 | Xenobiotics | 43 | 0.534884 | 0.162791 | Down | 0.005994 |
| 4810335 | Biosynthesis of the N-glycan precursor (dolichol lipid-linked oligosaccharide, LLO) and transfer to a nascent protein | 41 | 0.341463 | 0.04878 | Down | 0.005994 |
| 4810527 | Synthesis of bile acids and bile salts via 7alpha-hydroxycholesterol | 29 | 0.448276 | 0 | Down | 0.005994 |
| 4809933 | Cytosolic tRNA aminoacylation | 51 | 0.45098 | 0.078431 | Down | 0.005994 |
| 4809948 | Cap-dependent Translation Initiation | 182 | 0.120879 | 0.406593 | Up | 0.007992 |
| 4810795 | Regulation of Lipid Metabolism by Peroxisome proliferator-activated receptor alpha (PPARalpha) | 134 | 0.402985 | 0.104478 | Down | 0.007992 |
| 4810315 | Metabolism of porphyrins | 24 | 0.5 | 0.083333 | Down | 0.007992 |
| 4810089 | Cytochrome P450—arranged by substrate type | 98 | 0.469388 | 0.112245 | Down | 0.007992 |

**Table S3.** *Cont.*

| **PathID** | **Pathway** | **NGenes** | **PropDown** | **PropUp** | **Direction** | **p Value** |
| --- | --- | --- | --- | --- | --- | --- |
| 4810317 | Bile acid and bile salt metabolism | 44 | 0.409091 | 0.045455 | Down | 0.007992 |
| 4810610 | TCR signaling | 120 | 0.1 | 0.241667 | Up | 0.007992 |
| 4810310 | Intrinsic Pathway | 24 | 0.375 | 0 | Down | 0.007992 |
| 4810667 | Peptide hormone metabolism | 91 | 0.197802 | 0.076923 | Down | 0.007992 |
| 4811065 | Synthesis of PIPs at the Golgi membrane | 25 | 0.36 | 0 | Down | 0.007992 |
| 4809893 | Fatty acid, triacylglycerol, and ketone body metabolism | 240 | 0.35 | 0.104167 | Down | 0.00999 |
| 4810949 | TRAF6 mediated induction of TAK1 complex | 18 | 0 | 0.222222 | Up | 0.00999 |
| 4810684 | GABA synthesis, release, reuptake and degradation | 38 | 0.236842 | 0.026316 | Down | 0.00999 |
| 4810966 | Prolactin receptor signaling | 52 | 0.153846 | 0.019231 | Down | 0.00999 |

NGenes = Number of genes in pathway. PropUP = Proportion of genes UP (Higher in first group). PropDOWN = Proportions of genes DOWN (Lower in first group).

**Table S4.** Genes differentially expressed in the liver of offspring in the HH group compared with those in the LH group.

| **SystematicName** | **GeneSymbol** | **Fold Change** | ***p* Value** | **FDR** |
| --- | --- | --- | --- | --- |
| NM_026610 | Ndufb4 | 1.559229 | 1.55E-10 | 4.08E-06 |
| NM_010233 | Fn1 | −1.54971 | 3.18E-10 | 4.59E-06 |
| NM_001134646 | NA | 1.531096 | 2.67E-09 | 2.90E-05 |
| NM_018860 | Rpl41 | 1.525653 | 4.98E-09 | 3.21E-05 |
| NM_001037741 | Gpx4 | 1.60836 | 5.13E-09 | 3.21E-05 |
| NM_025983 | Atp5e | 1.635132 | 5.92E-09 | 3.21E-05 |
| NM_011387 | Slc10a1 | −2.18761 | 8.34E-09 | 3.79E-05 |
| NM_153055 | Sec63 | −1.53244 | 8.73E-09 | 3.79E-05 |
| NM_172838 | Slc16a12 | −1.80441 | 1.40E-08 | 5.05E-05 |
| NM_013899 | Timm10 | 1.564547 | 1.76E-08 | 5.87E-05 |
| NM_008361 | Il1b | 1.763098 | 2.08E-08 | 6.02E-05 |
| NM_013650 | S100a8 | 2.419705 | 2.69E-08 | 7.01E-05 |
| NM_008361 | Il1b | 1.772605 | 2.75E-08 | 7.01E-05 |
| NM_008361 | Il1b | 1.833775 | 3.09E-08 | 7.01E-05 |
| NM_010197 | Fgf1 | −1.68769 | 4.26E-08 | 8.23E-05 |
| NM_008361 | Il1b | 1.771463 | 4.67E-08 | 8.23E-05 |
| NM_015828 | Gne | −1.55569 | 4.96E-08 | 8.23E-05 |
| ENSMUST00000109036 | Gm6710 | 1.792504 | 5.12E-08 | 8.23E-05 |
| NM_009114 | S100a9 | 3.040645 | 6.15E-08 | 8.72E-05 |
| NM_172254 | Dph3 | 1.564085 | 6.21E-08 | 8.72E-05 |
| NM_008808 | Pdgfa | 1.598521 | 6.84E-08 | 8.72E-05 |
| NM_009945 | Cox7a2 | 1.688171 | 7.21E-08 | 8.72E-05 |
| NM_008361 | Il1b | 1.791117 | 8.03E-08 | 9.41E-05 |
| NM_008361 | Il1b | 1.751864 | 9.25E-08 | 0.0001 |
| NM_146948 | Olfr342 | −1.70804 | 9.48E-08 | 0.0001 |
| AK012052 | Rps19 | 1.512243 | 9.86E-08 | 0.000102 |
| NM_206537 | Cyp2c54 | −3.07993 | 1.32E-07 | 0.000124 |
| NM_013897 | Timm8b | 1.549049 | 1.32E-07 | 0.000124 |
| NM_207665 | Olfr1537 | −1.53947 | 1.44E-07 | 0.000131 |
| NM_145559 | Slc2a9 | −1.52958 | 1.45E-07 | 0.000131 |
| AK172225 | Slc25a51 | 1.71514 | 1.51E-07 | 0.000131 |
| NM_008361 | Il1b | 1.789586 | 1.79E-07 | 0.00015 |
| NR_027821 | 1810032O08Rik | 1.725148 | 2.50E-07 | 0.000184 |
| NM_008361 | Il1b | 1.725431 | 2.57E-07 | 0.000184 |
| NM_001033167 | Slc22a23 | −1.54525 | 2.84E-07 | 0.000196 |
| ENSMUST00000102977 | Hist1h4i | 1.669694 | 2.96E-07 | 0.000197 |
| NM_144845 | Ugt3a2 | −1.88957 | 3.82E-07 | 0.000224 |
| NM_020042 | Mocs1 | −1.54185 | 4.05E-07 | 0.000234 |
| ENSMUST00000131882 | Cpn1 | 1.748233 | 4.83E-07 | 0.000262 |
| NM_008455 | Klkb1 | −2.05992 | 5.12E-07 | 0.000272 |
| ENSMUST00000036360 | BC028528 | 1.702138 | 5.29E-07 | 0.000272 |
| NM_029295 | Cklf | 1.670694 | 5.56E-07 | 0.000278 |
| NM_009805 | Cflar | −1.5317 | 5.78E-07 | 0.000284 |

**Table S4.** *Cont.*

| **SystematicName** | **GeneSymbol** | **Fold Change** | ***p* Value** | **FDR** |
| --- | --- | --- | --- | --- |
| NM_025384 | Dnajc15 | 1.548408 | 6.08E-07 | 0.00029 |
| NM_153484 | Tef | −1.76057 | 6.29E-07 | 0.000293 |
| NM_145978 | Pdlim2 | 1.550724 | 6.74E-07 | 0.0003 |
| NM_001199307 | Etohi1 | 1.676172 | 6.78E-07 | 0.0003 |
| NM_013706 | Cd52 | 1.847411 | 7.33E-07 | 0.000318 |
| NM_028696 | Nabp1 | 1.811321 | 7.65E-07 | 0.000328 |
| NM_007609 | Casp4 | 1.857921 | 7.85E-07 | 0.000329 |
| NM_028260 | Immp1l | 1.544946 | 7.91E-07 | 0.000329 |
| NM_008361 | Il1b | 1.654422 | 7.99E-07 | 0.000329 |
| NM_025315 | Med21 | 1.873346 | 8.05E-07 | 0.000329 |
| NM_007883 | Dsg2 | −1.54299 | 8.66E-07 | 0.000345 |
| NM_197980 | Cox19 | 1.504442 | 8.81E-07 | 0.000347 |
| NM_001199307 | Etohi1 | 1.630942 | 1.05E-06 | 0.00039 |
| ENSMUST00000123265 | Gm14325 | 1.694836 | 1.15E-06 | 0.000417 |
| NM_010185 | Fcer1g | 1.616311 | 1.31E-06 | 0.000453 |
| AK013921 | Gm6710 | 1.577175 | 1.34E-06 | 0.000458 |
| NM_029344 | Acyp2 | 1.707442 | 1.42E-06 | 0.000467 |
| NM_146316 | Olfr726 | −1.63122 | 1.54E-06 | 0.0005 |
| NM_028807 | Exoc3l4 | 1.543304 | 1.56E-06 | 0.000502 |
| NM_001081957 | Wfdc17 | 2.219256 | 1.83E-06 | 0.000551 |
| NM_010197 | Fgf1 | −1.56974 | 1.86E-06 | 0.000557 |
| NM_023127 | Polr2k | 1.719763 | 1.91E-06 | 0.000563 |
| ENSMUST00000124229 | Prkd3 | −1.70923 | 2.02E-06 | 0.000577 |
| NM_027215 | Tmem147 | 1.50193 | 2.06E-06 | 0.000577 |
| NM_026632 | Rpa3 | 1.564575 | 2.09E-06 | 0.000582 |
| NM_009112 | S100a10 | 1.68587 | 2.17E-06 | 0.000596 |
| NM_001040395 | Nadk2 | −1.62253 | 2.45E-06 | 0.000644 |
| NM_021395 | Hyou1 | −2.76874 | 2.49E-06 | 0.000647 |
| NM_053113 | Ear11 | 1.673622 | 2.51E-06 | 0.000647 |
| NM_007796 | Ctla2a | 1.508292 | 2.52E-06 | 0.000647 |
| NM_008220 | Hbb-bt | 1.598293 | 2.54E-06 | 0.000647 |
| NM_001033167 | Slc22a23 | −1.51027 | 2.76E-06 | 0.000657 |
| ENSMUST00000084674 | 4732465J04Rik | 1.587088 | 2.78E-06 | 0.00066 |
| NM_009945 | Cox7a2 | 1.776464 | 2.99E-06 | 0.00069 |
| NM_026517 | Rpl22l1 | 1.767047 | 3.38E-06 | 0.000743 |
| NM_007900 | Ect2 | 1.520319 | 3.42E-06 | 0.000743 |
| NM_007969 | Wfdc18 | 1.557437 | 3.44E-06 | 0.000743 |
| NM_008646 | Mug2 | −2.00091 | 3.64E-06 | 0.000762 |
| NM_007534 | Bcl2a1b | 2.637982 | 3.93E-06 | 0.000797 |
| NM_010001 | Cyp2c37 | −2.71794 | 3.96E-06 | 0.0008 |
| NM_027959 | Pdia6 | −2.13389 | 4.22E-06 | 0.000825 |
| NM_144821 | AI317395 | −1.59666 | 4.31E-06 | 0.000835 |
| NM_008737 | Nrp1 | −2.0091 | 4.38E-06 | 0.000845 |
| NM_026418 | Rgs10 | 1.773718 | 4.45E-06 | 0.000846 |
| NM_008720 | Npc1 | −1.59337 | 4.53E-06 | 0.000852 |

**Table S4.** *Cont.*

| **SystematicName** | **GeneSymbol** | **Fold Change** | ***p* Value** | **FDR** |
| --- | --- | --- | --- | --- |
| NM_027840 | Snx20 | 1.574296 | 4.61E-06 | 0.000858 |
| NM_010654 | Klrd1 | 1.658943 | 4.84E-06 | 0.000894 |
| NM_013498 | Crem | 1.564472 | 5.11E-06 | 0.000932 |
| NM_008645 | Mug1 | −1.55536 | 5.25E-06 | 0.000952 |
| NM_008361 | Il1b | 1.599948 | 5.60E-06 | 0.000979 |
| NM_030691 | Igsf6 | 1.80355 | 5.66E-06 | 0.000979 |
| NM_031197 | Slc2a2 | −2.34365 | 5.72E-06 | 0.000979 |
| NM_013498 | Crem | 1.515028 | 5.72E-06 | 0.000979 |
| NM_008131 | Glul | −1.62628 | 5.77E-06 | 0.000979 |
| NM_009306 | Syt1 | −1.92683 | 5.81E-06 | 0.000979 |
| NM_030248 | Cdk5rap3 | −1.59026 | 5.85E-06 | 0.000979 |
| NM_027360 | 2010107E04Rik | 1.647153 | 6.02E-06 | 0.000996 |
| NM_013821 | Hsd3b6 | −1.71014 | 6.13E-06 | 0.001001 |
| NM_011631 | Hsp90b1 | −1.7069 | 6.19E-06 | 0.001006 |
| NM_027184 | Ipmk | −1.65657 | 6.77E-06 | 0.001064 |
| NM_011095 | Pirb | 1.722189 | 6.79E-06 | 0.001064 |
| NM_144800 | Mtss1 | −1.53812 | 6.81E-06 | 0.001064 |
| NM_030143 | Ddit4l | 1.523367 | 6.95E-06 | 0.001074 |
| ENSMUST00000160671 | Lss | −1.62455 | 7.62E-06 | 0.00114 |
| NM_028780 | Tm9sf1 | −1.5081 | 7.79E-06 | 0.001157 |
| NM_011817 | Gadd45g | 1.819439 | 7.94E-06 | 0.001167 |
| NM_026962 | Kbtbd3 | 1.524979 | 7.99E-06 | 0.001171 |
| NM_001099674 | 1810022K09Rik | 1.766953 | 8.60E-06 | 0.001229 |
| NM_011305 | Rxra | −1.53477 | 8.61E-06 | 0.001229 |
| NM_175188 | March1 | 1.56175 | 8.61E-06 | 0.001229 |
| NM_001109661 | Bach2 | −1.57456 | 8.91E-06 | 0.001264 |
| NM_153484 | Tef | −2.46102 | 8.96E-06 | 0.001264 |
| NM_145367 | Txndc5 | −1.55424 | 8.98E-06 | 0.001264 |
| NM_008877 | Plg | −1.6624 | 9.04E-06 | 0.001267 |
| NM_009722 | Atp2a2 | −1.70149 | 9.09E-06 | 0.001267 |
| NM_028608 | Glipr1 | 1.551202 | 9.16E-06 | 0.00127 |
| NM_001037863 | Atp11c | −1.51326 | 9.58E-06 | 0.00131 |
| NM_172943 | Alkbh5 | −1.62687 | 9.89E-06 | 0.001334 |
| NM_145509 | 5430435G22Rik | 1.68363 | 9.90E-06 | 0.001334 |
| NM_011521 | Sdc4 | −2.10222 | 9.96E-06 | 0.001336 |
| NM_021891 | Fignl1 | 1.537201 | 1.01E-05 | 0.001336 |
| NM_007815 | Cyp2c29 | −1.72859 | 1.07E-05 | 0.001383 |
| NM_001128151 | Cecr2 | −1.5578 | 1.08E-05 | 0.001383 |
| NM_011210 | Ptprc | 1.665298 | 1.08E-05 | 0.001384 |
| NM_025379 | Cox7b | 1.570188 | 1.12E-05 | 0.001414 |
| NM_027959 | Pdia6 | −1.4996 | 1.12E-05 | 0.001414 |
| NM_009245 | Serpina1c | −1.66112 | 1.15E-05 | 0.001421 |
| NM_134158 | AF251705 | 1.986073 | 1.16E-05 | 0.001421 |
| NM_009183 | St8sia4 | 1.515687 | 1.17E-05 | 0.001421 |

**Table S4.** *Cont.*

| **SystematicName** | **GeneSymbol** | **Fold Change** | ***p* Value** | **FDR** |
| --- | --- | --- | --- | --- |
| NM_176952 | 6430573F11Rik | 1.505361 | 1.18E-05 | 0.001421 |
| NM_015733 | Casp9 | −1.501 | 1.18E-05 | 0.001421 |
| NM_009539 | Zap70 | 1.894797 | 1.22E-05 | 0.001458 |
| NM_011210 | Ptprc | 1.638001 | 1.26E-05 | 0.001472 |
| NM_011888 | Ccl19 | 1.624314 | 1.27E-05 | 0.001478 |
| NM_201645 | Ugt1a1 | −2.25838 | 1.32E-05 | 0.001513 |
| NM_011210 | Ptprc | 1.627519 | 1.38E-05 | 0.001564 |
| NM_001037727 | Arhgap25 | 1.779745 | 1.40E-05 | 0.001566 |
| NM_134103 | Il1rap | −1.51088 | 1.48E-05 | 0.001633 |
| NM_008220 | Hbb-bt | 1.630221 | 1.49E-05 | 0.001633 |
| NM_030717 | Lactb | −1.62865 | 1.52E-05 | 0.001645 |
| NM_172928 | Dclk3 | −1.67756 | 1.68E-05 | 0.001773 |
| NM_015753 | Zeb2 | 1.560401 | 1.77E-05 | 0.001836 |
| NM_001040130 | Tmem141 | 1.639017 | 1.80E-05 | 0.001837 |
| NM_027249 | Tlcd2 | −1.74189 | 1.80E-05 | 0.001837 |
| NM_008677 | Ncf4 | 1.564921 | 1.80E-05 | 0.001837 |
| NM_183278 | Fam25c | 1.621071 | 1.88E-05 | 0.001884 |
| NM_029942 | Prelid2 | 1.790722 | 1.93E-05 | 0.001918 |
| NM_145822 | Cd3eap | 1.561492 | 2.03E-05 | 0.00199 |
| NM_022026 | Aqp9 | −2.24202 | 2.09E-05 | 0.002018 |
| NM_010956 | Ogdh | −1.61141 | 2.09E-05 | 0.002018 |
| NR_029475 | 4930581F22Rik | −1.56857 | 2.11E-05 | 0.002027 |
| NM_010444 | Nr4a1 | 1.970449 | 2.13E-05 | 0.00203 |
| NM_019792 | Cyp3a25 | −1.7219 | 2.15E-05 | 0.002044 |
| NM_001025566 | Chka | 1.721269 | 2.19E-05 | 0.002056 |
| NM_009856 | Cd83 | 1.736464 | 2.26E-05 | 0.002098 |
| NM_010191 | Fdft1 | −1.57276 | 2.26E-05 | 0.002098 |
| ENSMUST00000150975 | Pabpn1 | 1.553103 | 2.32E-05 | 0.002144 |
| NM_008408 | Stt3a | −1.78523 | 2.35E-05 | 0.002156 |
| NM_145634 | Cd300lf | 1.602387 | 2.42E-05 | 0.002192 |
| NM_027881 | Osbpl3 | 1.579119 | 2.44E-05 | 0.002194 |
| NM_009787 | Pdia4 | −2.10775 | 2.47E-05 | 0.002194 |
| NM_013652 | Ccl4 | 1.576823 | 2.48E-05 | 0.002194 |
| NM_080465 | Kcnn2 | −1.7369 | 2.56E-05 | 0.002223 |
| NM_010700 | Ldlr | −1.72864 | 2.59E-05 | 0.002234 |
| NM_011210 | Ptprc | 1.604717 | 2.59E-05 | 0.002234 |
| NM_027907 | Etnppl | 1.855164 | 2.60E-05 | 0.002239 |
| NM_011210 | Ptprc | 1.635512 | 2.66E-05 | 0.002255 |
| NM_008220 | Hbb-bt | 1.548071 | 2.67E-05 | 0.002255 |
| NM_010700 | Ldlr | −1.69453 | 2.67E-05 | 0.002255 |
| NM_007669 | Cdkn1a | 1.813284 | 2.68E-05 | 0.002255 |
| NM_144836 | Slc17a2 | −1.72646 | 2.70E-05 | 0.002263 |
| D86344 | Pdcd4 | −1.76764 | 2.79E-05 | 0.002316 |
| NM_011210 | Ptprc | 1.569359 | 2.87E-05 | 0.002368 |

**Table S4.** *Cont.*

| **SystematicName** | **GeneSymbol** | **Fold Change** | ***p* Value** | **FDR** |
| --- | --- | --- | --- | --- |
| NM_011210 | Ptprc | 1.577391 | 2.89E-05 | 0.00237 |
| NM_028780 | Tm9sf1 | −1.50648 | 2.94E-05 | 0.002397 |
| NM_029296 | 1700001C19Rik | −1.51448 | 2.94E-05 | 0.002397 |
| NM_008055 | Fzd4 | −1.51874 | 2.95E-05 | 0.002401 |
| NM_010700 | Ldlr | −1.69342 | 2.97E-05 | 0.002412 |
| NM_011333 | Ccl2 | 1.767903 | 3.01E-05 | 0.002432 |
| NM_008748 | Dusp8 | 1.606251 | 3.03E-05 | 0.002437 |
| NM_010700 | Ldlr | −1.72813 | 3.04E-05 | 0.002439 |
| NM_007403 | Adam8 | 1.961573 | 3.06E-05 | 0.002439 |
| NM_175654 | Hist1h4d | 1.575501 | 3.08E-05 | 0.002451 |
| NM_001035533 | Akap2 | 2.61018 | 3.16E-05 | 0.002493 |
| NM_011210 | Ptprc | 1.611805 | 3.19E-05 | 0.002505 |
| NM_008696 | Map4k4 | 1.53296 | 3.21E-05 | 0.002505 |
| NM_010742 | Ly6d | 4.72239 | 3.27E-05 | 0.00253 |
| NM_011210 | Ptprc | 1.581924 | 3.51E-05 | 0.002666 |
| NM_009963 | Cry2 | −1.65295 | 3.51E-05 | 0.002666 |
| NM_173762 | Cenpe | 1.584472 | 3.55E-05 | 0.002672 |
| NM_134152 | Lpxn | 1.536701 | 3.56E-05 | 0.002672 |
| NM_133898 | N4bp2l1 | −1.90109 | 3.61E-05 | 0.002685 |
| NM_178611 | Lair1 | 1.867078 | 3.72E-05 | 0.002715 |
| NM_009695 | Apoc2 | 1.672222 | 3.80E-05 | 0.002754 |
| NM_016721 | Iqgap1 | 1.505556 | 3.89E-05 | 0.00279 |
| NM_175111 | Hspbap1 | 1.573365 | 3.96E-05 | 0.002821 |
| NM_021282 | Cyp2e1 | −1.84702 | 3.98E-05 | 0.002831 |
| NM_153193 | Hsd3b2 | −1.62713 | 4.00E-05 | 0.002833 |
| NM_022331 | Herpud1 | −1.8139 | 4.11E-05 | 0.002897 |
| NM_001077514 | NA | −1.67773 | 4.32E-05 | 0.002995 |
| NM_008877 | Plg | −1.64614 | 4.45E-05 | 0.003049 |
| NM_026958 | Slirp | 1.640676 | 4.46E-05 | 0.003049 |
| ENSMUST00000039926 | Dusp8 | 1.520121 | 4.49E-05 | 0.003061 |
| NM_010071 | Dok2 | 1.533743 | 4.55E-05 | 0.003076 |
| NM_010819 | Clec4d | 1.590718 | 4.64E-05 | 0.003113 |
| NM_020008 | Clec7a | 3.01167 | 4.67E-05 | 0.003125 |
| ENSMUST00000125411 | Chka | 3.195361 | 4.73E-05 | 0.003149 |
| BC056964 | Taf1d | 1.557932 | 4.95E-05 | 0.003278 |
| NM_198092 | Usp2 | −2.17445 | 5.14E-05 | 0.003366 |
| NM_009171 | Shmt1 | −1.63749 | 5.15E-05 | 0.003366 |
| NM_145364 | Akr1d1 | −1.72195 | 5.21E-05 | 0.003371 |
| ENSMUST00000098499 | Ces1h | −1.67269 | 5.24E-05 | 0.003377 |
| NM_010700 | Ldlr | −1.67008 | 5.25E-05 | 0.003377 |
| NM_010559 | Il6ra | −1.54617 | 5.28E-05 | 0.003389 |
| NM_010700 | Ldlr | −1.67817 | 5.39E-05 | 0.003417 |
| NM_175090 | Slc31a1 | −1.55172 | 5.43E-05 | 0.003436 |
| NM_153392 | Ttc39a | 1.648456 | 5.52E-05 | 0.003475 |

**Table S4.** *Cont.*

| **SystematicName** | **GeneSymbol** | **Fold Change** | ***p* Value** | **FDR** |
| --- | --- | --- | --- | --- |
| NM_021366 | Klf13 | −1.63791 | 5.55E-05 | 0.003483 |
| ENSMUST00000023522 | Kalrn | −1.72102 | 5.57E-05 | 0.003484 |
| NM_010700 | Ldlr | −1.67459 | 5.59E-05 | 0.003491 |
| NM_010442 | Hmox1 | 1.568276 | 5.63E-05 | 0.003499 |
| NM_027209 | Ms4a6b | 1.847205 | 5.80E-05 | 0.003552 |
| AK020313 | 9230104K21Rik | 1.75161 | 5.83E-05 | 0.003563 |
| NM_008280 | Lipc | −1.77041 | 5.92E-05 | 0.003594 |
| NM_173047 | Cbr3 | 1.710686 | 5.95E-05 | 0.003604 |
| NM_010118 | Egr2 | 1.546223 | 6.03E-05 | 0.003642 |
| NM_007669 | Cdkn1a | 1.795793 | 6.21E-05 | 0.003705 |
| NM_008495 | Lgals1 | 1.851569 | 6.38E-05 | 0.003795 |
| NM_025436 | Sc4mol | −1.73056 | 6.43E-05 | 0.003814 |
| NM_017379 | Tuba8 | 1.825828 | 6.44E-05 | 0.003814 |
| NM_153484 | Tef | −1.4993 | 6.48E-05 | 0.003835 |
| NM_011978 | Slc27a2 | −1.53976 | 6.53E-05 | 0.003855 |
| NM_010700 | Ldlr | −1.66799 | 6.64E-05 | 0.003888 |
| NR_028575 | 0610007N19Rik | 1.59343 | 6.68E-05 | 0.003897 |
| NR_001460 | Rmrp | 2.16371 | 6.69E-05 | 0.003903 |
| NM_175417 | Adtrp | −1.60082 | 6.80E-05 | 0.003908 |
| NM_012030 | Slc9a3r1 | −1.58592 | 6.88E-05 | 0.003924 |
| NM_011210 | Ptprc | 1.589516 | 6.88E-05 | 0.003924 |
| NM_028071 | Cotl1 | 1.597858 | 6.93E-05 | 0.003926 |
| NM_001033450 | Mnda | 1.54998 | 6.93E-05 | 0.003926 |
| NM_010700 | Ldlr | −1.71807 | 7.05E-05 | 0.003977 |
| NM_022024 | Gmfg | 1.513209 | 7.06E-05 | 0.003977 |
| NM_019776 | Snd1 | −1.76471 | 7.08E-05 | 0.003982 |
| NM_008745 | Ntrk2 | 2.997651 | 7.12E-05 | 0.003993 |
| NM_009227 | Snrpe | 1.574256 | 7.62E-05 | 0.004146 |
| NM_023223 | Cdc20 | 1.960533 | 7.83E-05 | 0.0042 |
| NM_001033302 | Gm129 | −2.85274 | 7.87E-05 | 0.004209 |
| NM_001033711 | Evi2a | 1.652765 | 7.89E-05 | 0.004216 |
| NM_146245 | Lrit1 | −1.61638 | 8.08E-05 | 0.004277 |
| NM_008745 | Ntrk2 | 2.939008 | 8.21E-05 | 0.004308 |
| NM_010956 | Ogdh | −1.67441 | 8.21E-05 | 0.004308 |
| NM_010233 | Fn1 | −2.34195 | 8.48E-05 | 0.004402 |
| NM_008745 | Ntrk2 | 2.998979 | 8.50E-05 | 0.004402 |
| NM_001039354 | Lin7a | −1.51306 | 8.73E-05 | 0.004466 |
| NM_178611 | Lair1 | 1.738112 | 8.82E-05 | 0.004498 |
| NM_133774 | Stard4 | −1.52425 | 8.86E-05 | 0.004508 |
| NM_023184 | Klf15 | −1.93316 | 8.88E-05 | 0.004508 |
| ENSMUST00000000153 | NA | −1.58297 | 8.95E-05 | 0.004531 |
| NM_175687 | A230050P20Rik | 1.675931 | 9.05E-05 | 0.004569 |
| NM_010233 | Fn1 | −1.99961 | 9.28E-05 | 0.004654 |
| NM_019972 | Sort1 | −1.51362 | 9.40E-05 | 0.004699 |

**Table S4.** *Cont.*

| **SystematicName** | **GeneSymbol** | **Fold Change** | ***p* Value** | **FDR** |
| --- | --- | --- | --- | --- |
| NM_010705 | Lgals3 | 1.613419 | 9.49E-05 | 0.004727 |
| NM_008745 | Ntrk2 | 2.921605 | 9.68E-05 | 0.004764 |
| NM_028233 | Lrpprc | −1.7046 | 9.81E-05 | 0.004792 |
| ENSMUST00000092163 | Lyz2 | 1.966589 | 9.82E-05 | 0.004792 |
| NM_175177 | Bdh1 | −1.56177 | 9.96E-05 | 0.004828 |
| NM_012019 | Aifm1 | −2.01531 | 9.98E-05 | 0.004832 |
| NM_022026 | Aqp9 | −2.08621 | 0.000101 | 0.004856 |
| NM_133960 | Ces2a | −1.59115 | 0.000101 | 0.004865 |
| NM_033596 | Hist2h4 | 1.542042 | 0.000102 | 0.004905 |
| NM_009255 | Serpine2 | −1.68474 | 0.000104 | 0.004958 |
| NM_146489 | Olfr266 | −1.53117 | 0.000104 | 0.004964 |
| NM_010565 | Inhbc | −1.68734 | 0.000105 | 0.004981 |
| NM_027052 | Slc38a4 | −1.78079 | 0.000105 | 0.004991 |
| NM_134072 | Akr1c14 | −1.50199 | 0.000106 | 0.004995 |
| NM_013470 | Anxa3 | 1.679411 | 0.000106 | 0.004997 |
| NM_015828 | Gne | −1.54846 | 0.000106 | 0.005014 |
| NM_010196 | Fga | −1.65606 | 0.000107 | 0.005038 |
| NM_011333 | Ccl2 | 1.70463 | 0.000111 | 0.005123 |
| NM_001037724 | Adcy7 | 1.520709 | 0.000111 | 0.005123 |
| NM_028222 | Cdkn3 | 1.802952 | 0.000112 | 0.005154 |
| NM_008087 | Gas2 | 1.608401 | 0.000112 | 0.005154 |
| NM_001079695 | Srsf5 | −1.83221 | 0.000113 | 0.005154 |
| NM_010212 | Fhl2 | 1.885629 | 0.000114 | 0.005167 |
| NM_008745 | Ntrk2 | 2.97074 | 0.000114 | 0.005175 |
| NM_010233 | Fn1 | −1.97882 | 0.000114 | 0.005188 |
| NM_010233 | Fn1 | −1.91809 | 0.000115 | 0.005201 |
| NM_153800 | Arhgap22 | 1.580805 | 0.000116 | 0.005218 |
| NM_010559 | Il6ra | −1.59261 | 0.000117 | 0.005233 |
| NR_027059 | 2810008D09Rik | 1.519846 | 0.000118 | 0.005264 |
| NM_008745 | Ntrk2 | 2.889994 | 0.00012 | 0.005318 |
| NM_010233 | Fn1 | −1.95919 | 0.000121 | 0.005336 |
| NM_053262 | Hsd17b11 | −1.65944 | 0.000121 | 0.005336 |
| NM_030026 | Mccc2 | −1.64507 | 0.000124 | 0.005444 |
| NM_008745 | Ntrk2 | 2.979774 | 0.000125 | 0.005449 |
| NM_008745 | Ntrk2 | 2.881293 | 0.000126 | 0.005475 |
| NM_010233 | Fn1 | −1.95332 | 0.000126 | 0.005475 |
| NM_009915 | Ccr2 | 1.535164 | 0.000129 | 0.005551 |
| NM_007669 | Cdkn1a | 1.782951 | 0.000129 | 0.005558 |
| NM_145603 | Ces2c | −1.61242 | 0.000129 | 0.005569 |
| NM_008642 | Mttp | −1.5128 | 0.000132 | 0.005625 |
| NM_007643 | Cd36 | 1.833468 | 0.000133 | 0.005657 |
| ENSMUST00000128428 | Frmd5 | 1.779755 | 0.000133 | 0.005657 |
| NM_011333 | Ccl2 | 1.712794 | 0.000134 | 0.005657 |
| NM_010233 | Fn1 | −1.95918 | 0.000134 | 0.005667 |

**Table S4.** *Cont.*

| **SystematicName** | **GeneSymbol** | **Fold Change** | ***p* Value** | **FDR** |
| --- | --- | --- | --- | --- |
| NM_008823 | Cfp | 1.499663 | 0.000134 | 0.005667 |
| NM_008745 | Ntrk2 | 2.84531 | 0.000135 | 0.00568 |
| NM_011333 | Ccl2 | 1.692559 | 0.000136 | 0.005697 |
| NM_009656 | Aldh2 | −1.68992 | 0.000143 | 0.005932 |
| NM_175836 | Sptbn1 | 1.576138 | 0.000145 | 0.005989 |
| NM_007669 | Cdkn1a | 1.772223 | 0.000146 | 0.005989 |
| NM_009730 | Atrn | −1.50263 | 0.000149 | 0.006072 |
| NM_028295 | Pdia5 | −1.52387 | 0.00015 | 0.006087 |
| NM_007669 | Cdkn1a | 1.718014 | 0.000152 | 0.006125 |
| NM_007669 | Cdkn1a | 1.705253 | 0.000154 | 0.006179 |
| NM_010118 | Egr2 | 1.501414 | 0.000155 | 0.006214 |
| NM_010233 | Fn1 | −1.94622 | 0.000157 | 0.006259 |
| NM_010118 | Egr2 | 1.563951 | 0.000159 | 0.006273 |
| NM_015763 | Lpin1 | −2.27325 | 0.00016 | 0.006288 |
| NM_023158 | Cxcl16 | 1.646145 | 0.00016 | 0.006288 |
| NM_011333 | Ccl2 | 1.69647 | 0.00016 | 0.006288 |
| NM_025834 | Proz | −1.54436 | 0.00016 | 0.006288 |
| NM_008995 | Pex5 | −1.56602 | 0.000163 | 0.006333 |
| NM_008058 | Fzd8 | −1.53729 | 0.000163 | 0.006333 |
| NM_010497 | Idh1 | −1.57631 | 0.000165 | 0.006368 |
| NM_013623 | Orm3 | 1.830655 | 0.000171 | 0.006566 |
| NM_172671 | Lgr4 | −1.49878 | 0.000175 | 0.006624 |
| NM_011316 | Saa4 | −1.67499 | 0.00018 | 0.00673 |
| NM_013754 | Insl6 | 1.613274 | 0.000183 | 0.006768 |
| NM_029623 | 3110002H16Rik | −1.50224 | 0.000184 | 0.006786 |
| NM_001025610 | Ms4a7 | 2.007647 | 0.00019 | 0.006921 |
| NM_009647 | Ak4 | −1.60225 | 0.00019 | 0.006922 |
| NM_173405 | Amz1 | 1.501152 | 0.00019 | 0.006922 |
| NM_010700 | Ldlr | −1.6468 | 0.00019 | 0.006922 |
| NM_008353 | Il12rb1 | −1.58769 | 0.000191 | 0.006936 |
| NM_007669 | Cdkn1a | 1.748985 | 0.000191 | 0.006936 |
| NM_008745 | Ntrk2 | 2.796522 | 0.000191 | 0.006936 |
| NM_027218 | Clec4b1 | 1.839943 | 0.000195 | 0.007016 |
| NM_001012306 | Hsd3b3 | −1.70336 | 0.000195 | 0.007019 |
| NM_182959 | Slc17a8 | −2.45498 | 0.000196 | 0.007019 |
| NM_007669 | Cdkn1a | 1.736293 | 0.000197 | 0.007049 |
| NM_028472 | Bmper | 1.664201 | 0.000197 | 0.007055 |
| NM_001081359 | Ubr5 | −1.68101 | 0.000198 | 0.007064 |
| NM_177322 | Agtr1a | −1.58622 | 0.000198 | 0.007064 |
| NM_053110 | Gpnmb | 2.644516 | 0.000199 | 0.007064 |
| NM_024282 | Desi2 | 1.591982 | 0.0002 | 0.007086 |
| NM_025520 | Lsm5 | 1.532014 | 0.000202 | 0.007107 |
| NM_134469 | Fdps | −1.79008 | 0.000206 | 0.007201 |
| NM_007669 | Cdkn1a | 1.700674 | 0.000206 | 0.007201 |

**Table S4.** *Cont.*

| **SystematicName** | **GeneSymbol** | **Fold Change** | ***p* Value** | **FDR** |
| --- | --- | --- | --- | --- |
| NM_010233 | Fn1 | −1.91584 | 0.000207 | 0.007207 |
| NM_019467 | Aif1 | 1.575818 | 0.000207 | 0.007216 |
| NM_080563 | Rnf144a | 1.516218 | 0.00021 | 0.007255 |
| NM_001004147 | Fbll1 | −1.51064 | 0.00021 | 0.007261 |
| NM_001025610 | Ms4a7 | 1.897575 | 0.000212 | 0.007291 |
| NM_009139 | Ccl6 | 1.559183 | 0.000213 | 0.007323 |
| NM_028173 | Tram1 | −1.56034 | 0.000213 | 0.007323 |
| NM_009804 | Cat | −1.50381 | 0.000219 | 0.00743 |
| NM_178378 | Iqcg | 1.518952 | 0.00022 | 0.007438 |
| NM_007636 | Cct2 | −1.58304 | 0.000223 | 0.007513 |
| NM_010233 | Fn1 | −1.93287 | 0.000225 | 0.007567 |
| NM_011333 | Ccl2 | 1.60325 | 0.00023 | 0.007687 |
| NM_007669 | Cdkn1a | 1.690211 | 0.000231 | 0.007721 |
| NM_183262 | Stk35 | −1.60037 | 0.00024 | 0.007932 |
| NM_010233 | Fn1 | −1.94459 | 0.000242 | 0.007959 |
| NM_007981 | Acsl1 | −1.54771 | 0.000243 | 0.007971 |
| NR_002847 | Malat1 | 2.141006 | 0.000244 | 0.007991 |
| NM_008991 | Abcd3 | −1.86658 | 0.000248 | 0.008062 |
| NM_024459 | Ppp3r1 | −1.61987 | 0.000248 | 0.008064 |
| NM_008197 | H1f0 | −1.80205 | 0.000251 | 0.008123 |
| NM_029692 | Upp2 | −2.69345 | 0.000255 | 0.008211 |
| NM_173788 | Npr2 | −1.70232 | 0.000256 | 0.008236 |
| NM_018869 | Grk5 | 1.592997 | 0.000258 | 0.008283 |
| NM_019641 | Stmn1 | 1.578669 | 0.000258 | 0.008283 |
| NM_019769 | Chp1 | −1.8788 | 0.000261 | 0.008343 |
| NM_145564 | Fbxo21 | −1.74903 | 0.000267 | 0.008438 |
| NM_001162999 | Fnip2 | −1.51053 | 0.00027 | 0.008492 |
| NM_011662 | Tyrobp | 1.771844 | 0.000271 | 0.008525 |
| NM_175189 | Hepacam | 1.52696 | 0.000271 | 0.008527 |
| NM_145449 | Ifi27l2b | 1.803646 | 0.000274 | 0.008567 |
| NM_019992 | Stap1 | 1.555863 | 0.000284 | 0.008804 |
| NM_145130 | Lpcat3 | −1.54744 | 0.000298 | 0.009075 |
| AK030395 | Etnppl | 2.249683 | 0.000303 | 0.009173 |
| NM_145434 | Nr1d1 | −1.6856 | 0.000303 | 0.009173 |
| NM_007559 | Bmp8b | 1.663895 | 0.000317 | 0.009454 |
| NM_172606 | Mar-06 | −1.53977 | 0.000319 | 0.009461 |
| NM_011704 | Vnn1 | −1.69558 | 0.000322 | 0.009524 |
| NM_011333 | Ccl2 | 1.644309 | 0.000326 | 0.009568 |
| NM_011388 | Slc10a2 | 1.5738 | 0.000335 | 0.009725 |
| ENSMUST00000133968 | Rasgrp2 | 1.663306 | 0.000337 | 0.009752 |
| NM_029720 | Creld2 | −1.92341 | 0.000343 | 0.009906 |
| NM_011575 | Tff3 | 1.568695 | 0.000355 | 0.010096 |
| NM_021281 | Ctss | 1.765872 | 0.000356 | 0.010108 |
| NM_016854 | Ppp1r3c | 1.782444 | 0.000357 | 0.01011 |

**Table S4.** *Cont.*

| **SystematicName** | **GeneSymbol** | **Fold Change** | ***p* Value** | **FDR** |
| --- | --- | --- | --- | --- |
| NM_010638 | Klf9 | −1.59144 | 0.000357 | 0.010121 |
| NM_009899 | Clca1 | 1.593368 | 0.000363 | 0.010238 |
| NM_011144 | Ppara | −1.7044 | 0.000366 | 0.010277 |
| NM_016974 | Dbp | −4.27526 | 0.000367 | 0.010299 |
| NM_029347 | Fggy | −1.55726 | 0.000368 | 0.010318 |
| NM_009306 | Syt1 | −1.61607 | 0.00037 | 0.010352 |
| NM_133217 | Bco2 | −1.60742 | 0.00037 | 0.010367 |
| NM_024264 | Cyp27a1 | −1.70545 | 0.000376 | 0.010464 |
| NM_011825 | Grem2 | −1.68807 | 0.000376 | 0.010464 |
| NM_013590 | Lyz1 | 1.986335 | 0.000382 | 0.010573 |
| NR_002847 | Malat1 | 2.792185 | 0.00039 | 0.010716 |
| NM_027016 | Sec62 | −1.57474 | 0.000392 | 0.010728 |
| NM_025834 | Proz | −1.53908 | 0.000392 | 0.010728 |
| NM_027309 | Lysmd2 | 1.552366 | 0.000392 | 0.010728 |
| NM_011144 | Ppara | −1.70449 | 0.0004 | 0.010873 |
| NM_145367 | Txndc5 | −1.53279 | 0.000411 | 0.01107 |
| NM_023066 | Asph | −1.51463 | 0.000417 | 0.011171 |
| ENSMUST00000054128 | Dsg1c | −1.89415 | 0.00042 | 0.011219 |
| NM_001037221 | Samd4 | 1.534519 | 0.00042 | 0.011228 |
| NM_008529 | Ly6e | 1.551902 | 0.000425 | 0.011293 |
| NM_207655 | Egfr | −1.97287 | 0.000431 | 0.01138 |
| NM_023281 | Sdha | −1.56447 | 0.000432 | 0.011381 |
| NM_133834 | Hnrnpf | −1.70774 | 0.000443 | 0.011571 |
| NM_022324 | Sdf2l1 | −1.98058 | 0.000444 | 0.011602 |
| NM_153526 | Insig1 | −1.93197 | 0.000445 | 0.011614 |
| NM_173788 | Npr2 | −1.57336 | 0.000449 | 0.011688 |
| NM_153164 | Cnot1 | −1.54927 | 0.000452 | 0.011741 |
| NM_001039710 | Coq10b | −1.69379 | 0.000459 | 0.011827 |
| NM_021272 | Fabp7 | 1.668325 | 0.000461 | 0.011854 |
| NM_175687 | NA | 1.610229 | 0.000481 | 0.012149 |
| NM_013749 | Tnfrsf12a | 2.096028 | 0.000484 | 0.012181 |
| NM_025279 | Hnrnpk | −1.61998 | 0.000492 | 0.012311 |
| NM_011333 | Ccl2 | 1.634521 | 0.000501 | 0.012465 |
| NM_011333 | Ccl2 | 1.551192 | 0.000522 | 0.01277 |
| NM_145564 | Fbxo21 | −1.60013 | 0.000539 | 0.013067 |
| NM_023137 | Ubd | 1.611211 | 0.000551 | 0.013251 |
| NM_007535 | Bcl2a1c | 1.588616 | 0.000551 | 0.013254 |
| NM_011144 | Ppara | −1.68128 | 0.000558 | 0.013345 |
| NM_201375 | Kng2 | −1.63819 | 0.000573 | 0.013556 |
| NM_010011 | Cyp4a10 | −1.86836 | 0.000575 | 0.013585 |
| NM_207655 | Egfr | −1.96031 | 0.000576 | 0.013592 |
| NM_007618 | Serpina6 | −1.66857 | 0.000595 | 0.013837 |
| NM_026221 | Ppfibp1 | 1.532845 | 0.000599 | 0.013888 |
| NM_178617 | Necab1 | −1.84165 | 0.000603 | 0.01395 |

**Table S4.** *Cont.*

| **SystematicName** | **GeneSymbol** | **Fold Change** | ***p* Value** | **FDR** |
| --- | --- | --- | --- | --- |
| NM_011144 | Ppara | −1.68424 | 0.000606 | 0.013983 |
| ENSMUST00000126850 | Ccnt2 | 1.498829 | 0.000609 | 0.014003 |
| NM_015828 | Gne | −1.61495 | 0.000631 | 0.014377 |
| NM_022722 | Dpys | −1.56711 | 0.000645 | 0.014546 |
| NM_019911 | Tdo2 | −1.63819 | 0.000647 | 0.014571 |
| NM_207655 | Egfr | −1.96167 | 0.000649 | 0.014605 |
| NM_007606 | Car3 | −1.99801 | 0.000657 | 0.014734 |
| NM_024440 | Derl3 | −3.28154 | 0.000658 | 0.014734 |
| NR_002847 | Malat1 | 1.699124 | 0.000669 | 0.014914 |
| NM_011144 | Ppara | −1.66705 | 0.000688 | 0.015149 |
| NM_201640 | Cyp4a31 | −1.90476 | 0.000688 | 0.015149 |
| NM_019769 | Chp1 | −1.87933 | 0.000692 | 0.015195 |
| NM_133903 | Spon2 | 1.771957 | 0.000696 | 0.01527 |
| NM_007833 | Dcn | 1.612768 | 0.000698 | 0.015286 |
| NM_207655 | Egfr | −1.9579 | 0.000718 | 0.015583 |
| AK075966 | D17H6S56E-5 | 1.53066 | 0.00073 | 0.015755 |
| NM_029612 | Slamf9 | 1.549438 | 0.00074 | 0.015868 |
| NM_207655 | Egfr | −1.9404 | 0.000744 | 0.015917 |
| NM_026027 | Pfdn1 | 1.544346 | 0.000748 | 0.015975 |
| NM_207655 | Egfr | −1.93697 | 0.000756 | 0.016097 |
| NM_207655 | Egfr | −1.92111 | 0.000758 | 0.016116 |
| NM_011267 | Rgs16 | −2.80549 | 0.000777 | 0.016349 |
| ENSMUST00000000153 | NA | −1.74472 | 0.000784 | 0.016436 |
| NM_207655 | Egfr | −1.94452 | 0.000785 | 0.016436 |
| BC029734 | Fgl1 | −1.52453 | 0.000789 | 0.016504 |
| NM_017381 | Zranb2 | 1.5766 | 0.000795 | 0.016587 |
| NM_011461 | Spic | 1.513291 | 0.000797 | 0.016611 |
| NM_020001 | Clec4n | 1.541943 | 0.000817 | 0.01687 |
| NM_013809 | Cyp2g1 | −1.6016 | 0.000828 | 0.017001 |
| NM_019911 | Tdo2 | −1.54786 | 0.000834 | 0.017065 |
| NM_015763 | Lpin1 | −2.02396 | 0.000837 | 0.017081 |
| NM_011144 | Ppara | −1.63517 | 0.000844 | 0.017166 |
| NM_009744 | Bcl6 | −2.38974 | 0.000855 | 0.01733 |
| NM_007812 | Cyp2a5 | −1.69271 | 0.000857 | 0.017349 |
| NM_011144 | Ppara | −1.63971 | 0.00086 | 0.017373 |
| NM_207655 | Egfr | −1.91295 | 0.000864 | 0.017428 |
| NM_011144 | Ppara | −1.64588 | 0.000883 | 0.017704 |
| NM_133903 | Spon2 | 1.630584 | 0.000889 | 0.017774 |
| NM_030026 | Mccc2 | −1.66382 | 0.000892 | 0.017797 |
| NM_031254 | Trem2 | 2.599099 | 0.000902 | 0.017936 |
| NM_207655 | Egfr | −1.93432 | 0.000913 | 0.018077 |
| NM_146126 | Sord | −1.6045 | 0.000924 | 0.018202 |
| NM_007742 | Col1a1 | 1.674251 | 0.000927 | 0.018261 |
| NM_013842 | Xbp1 | −1.65991 | 0.000936 | 0.018364 |

**Table S4.** *Cont.*

| **SystematicName** | **GeneSymbol** | **Fold Change** | ***p* Value** | **FDR** |
| --- | --- | --- | --- | --- |
| NM_010186 | Fcgr1 | 1.551624 | 0.000936 | 0.018364 |
| NM_008991 | Abcd3 | −1.82618 | 0.000939 | 0.018405 |
| ENSMUST00000123151 | Fus | 1.747007 | 0.000966 | 0.01875 |
| NM_207202 | Ccdc120 | 1.50163 | 0.000969 | 0.018769 |
| NM_027493 | Actr8 | −1.51372 | 0.000996 | 0.0191 |
| ENSMUST00000055719 | BC025446 | −1.57977 | 0.000998 | 0.019108 |
| NM_007836 | Gadd45a | −1.51048 | 0.001007 | 0.019243 |
| NM_015763 | Lpin1 | −1.99061 | 0.001015 | 0.019365 |
| NM_199029 | Zfp395 | −1.51621 | 0.001017 | 0.019365 |
| NM_207655 | Egfr | −1.96144 | 0.001028 | 0.019489 |
| NM_027237 | 2010003K11Rik | 2.238587 | 0.001029 | 0.01951 |
| NM_029331 | NA | −1.71619 | 0.00103 | 0.019515 |
| NR_028331 | Zfp672 | 1.646695 | 0.00104 | 0.019633 |
| NM_145980 | 8430408G22Rik | −2.29879 | 0.001042 | 0.01966 |
| NM_011144 | Ppara | −1.68259 | 0.00105 | 0.019751 |
| L23108 | Cd36 | 1.691417 | 0.001058 | 0.019855 |
| NM_007976 | F5 | −1.68565 | 0.001062 | 0.019886 |
| NM_025794 | Etfdh | −1.7175 | 0.001073 | 0.020031 |
| NM_015729 | Acox1 | −2.24787 | 0.001083 | 0.020131 |
| NM_010745 | Ly86 | 1.693633 | 0.001093 | 0.020257 |
| NM_008137 | Gna14 | −1.5009 | 0.001122 | 0.020582 |
| NM_026184 | Ero1lb | −1.62244 | 0.001133 | 0.020699 |
| NM_011333 | Ccl2 | 1.564867 | 0.001162 | 0.020983 |
| NM_009450 | Tubb2a | 1.68705 | 0.001195 | 0.021311 |
| NM_007742 | Col1a1 | 1.643923 | 0.001195 | 0.021311 |
| NM_025939 | Paics | −1.5817 | 0.001226 | 0.021672 |
| NM_027711 | Iqgap2 | −1.57612 | 0.00123 | 0.021708 |
| NM_001033988 | Ncoa4 | −1.53485 | 0.001258 | 0.021955 |
| NM_025827 | Lonp2 | −2.00808 | 0.001275 | 0.022124 |
| NM_013490 | Chka | 1.847429 | 0.001291 | 0.022252 |
| NM_027711 | Iqgap2 | −1.6655 | 0.001295 | 0.022283 |
| NM_007742 | Col1a1 | 1.666135 | 0.001316 | 0.022531 |
| NM_008908 | Ppic | 1.660324 | 0.001342 | 0.022774 |
| NM_007861 | Dld | −1.64392 | 0.001361 | 0.022946 |
| NM_053096 | Cml2 | −1.61189 | 0.001375 | 0.023129 |
| NM_025841 | Kdelr2 | −1.84305 | 0.001392 | 0.023249 |
| NM_175523 | Ppm1k | −1.59508 | 0.001412 | 0.023396 |
| NM_031254 | Trem2 | 1.954918 | 0.001423 | 0.023497 |
| NM_009892 | Chi3l3 | 1.943259 | 0.001427 | 0.023524 |
| NM_011065 | Per1 | −1.8921 | 0.001439 | 0.023642 |
| ENSMUST00000015595 | NA | −1.61693 | 0.001449 | 0.023751 |
| NM_001164885 | Lpin2 | −1.63939 | 0.001466 | 0.023956 |
| NM_008687 | Nfib | −1.66673 | 0.001479 | 0.024099 |
| NM_021334 | Itgax | 1.530223 | 0.001487 | 0.024162 |

**Table S4.** *Cont.*

| **SystematicName** | **GeneSymbol** | **Fold Change** | ***p* Value** | **FDR** |
| --- | --- | --- | --- | --- |
| NM_175250 | 2810007J24Rik | −1.5433 | 0.0015 | 0.024252 |
| NAP065150-1 | NA | −1.73839 | 0.001509 | 0.024305 |
| NM_008112 | Gdi2 | −1.55415 | 0.001514 | 0.024309 |
| ENSMUST00000141060 | Ttc39c | −1.50562 | 0.001516 | 0.024327 |
| NR_027939 | NA | 1.549181 | 0.001543 | 0.024581 |
| NM_022026 | Aqp9 | −1.71934 | 0.001551 | 0.024629 |
| NM_023125 | Kng1 | −1.77915 | 0.001562 | 0.024727 |
| NM_011920 | Abcg2 | −1.57328 | 0.001571 | 0.024826 |
| NM_013584 | Lifr | −1.57549 | 0.001589 | 0.024965 |
| NM_033354 | Sec16b | −1.62995 | 0.001604 | 0.025085 |
| NM_007742 | Col1a1 | 1.614989 | 0.001606 | 0.025085 |
| NM_022435 | Sp5 | −1.5008 | 0.001618 | 0.02521 |
| NM_198899 | Uggt1 | −1.51999 | 0.001664 | 0.025594 |
| NM_021278 | Tmsb4x | 1.844296 | 0.001684 | 0.025756 |
| NM_011631 | Hsp90b1 | −1.65954 | 0.001686 | 0.025756 |
| NM_019932 | Pf4 | 1.526635 | 0.00172 | 0.026063 |
| NM_028053 | Tmem38b | −1.62655 | 0.00173 | 0.026186 |
| NM_007742 | Col1a1 | 1.638886 | 0.001733 | 0.026211 |
| NM_133726 | St13 | −1.522 | 0.00175 | 0.026397 |
| NM_173864 | Gm5077 | −1.59463 | 0.001762 | 0.026491 |
| NM_178747 | Gulo | −1.59431 | 0.001787 | 0.026735 |
| AK137700 | Slc27a1 | −1.58927 | 0.001829 | 0.027053 |
| ENSMUST00000128133 | Rdh9 | −1.62525 | 0.001842 | 0.027154 |
| NM_008721 | Npdc1 | 1.520044 | 0.001846 | 0.027187 |
| NM_009266 | Sephs2 | −1.65498 | 0.001859 | 0.027246 |
| NM_138745 | Mthfd1 | −1.6359 | 0.001859 | 0.027246 |
| NM_009723 | Atp2b2 | 2.792544 | 0.0019 | 0.027597 |
| NM_031163 | Col2a1 | 1.520586 | 0.001941 | 0.027932 |
| NM_011048 | NA | −1.7701 | 0.00196 | 0.028071 |
| ENSMUST00000056153 | Fads6 | −1.6565 | 0.001964 | 0.028083 |
| NM_134101 | Psmd2 | −1.5527 | 0.001973 | 0.028173 |
| AJ539223 | Erdr1 | 1.525181 | 0.002023 | 0.028584 |
| NM_009690 | Cd5l | 1.579473 | 0.002029 | 0.028627 |
| NM_007742 | Col1a1 | 1.629296 | 0.002033 | 0.028638 |
| NM_178082 | Insig2 | 1.508009 | 0.002038 | 0.028682 |
| NM_007825 | Cyp7b1 | −2.27226 | 0.002072 | 0.028897 |
| NM_009061 | Rgs2 | 1.575463 | 0.002108 | 0.029238 |
| NM_007376 | Pzp | −1.54469 | 0.002114 | 0.029294 |
| NM_011095 | Pirb | 1.747059 | 0.002115 | 0.029294 |
| NM_152234 | Ubqln1 | −1.4995 | 0.002127 | 0.029411 |
| TC1682954 | NA | 1.668623 | 0.00219 | 0.029984 |
| ENSMUST00000123238 | Cfh | −1.61545 | 0.002242 | 0.030484 |
| NM_017372 | Lyz2 | 1.679518 | 0.002256 | 0.030616 |
| NM_010004 | Cyp2c40 | −1.49911 | 0.002257 | 0.030616 |

**Table S4.** *Cont.*

| **SystematicName** | **GeneSymbol** | **Fold Change** | ***p* Value** | **FDR** |
| --- | --- | --- | --- | --- |
| NM_153069 | Leap2 | 1.66928 | 0.002314 | 0.031066 |
| NM_011394 | Slc20a2 | −1.70226 | 0.002341 | 0.031286 |
| NM_007742 | Col1a1 | 1.617076 | 0.002358 | 0.031436 |
| NM_008294 | Hsd3b4 | −3.3649 | 0.002391 | 0.031763 |
| NM_019688 | Rapgef4 | −1.66569 | 0.002428 | 0.032065 |
| NM_011660 | Txn1 | 1.591993 | 0.002434 | 0.032115 |
| NM_015786 | Hist1h1c | 1.508843 | 0.002456 | 0.032293 |
| NM_023377 | Stard5 | −1.51789 | 0.002463 | 0.032346 |
| NM_178591 | Nrg1 | 1.544218 | 0.002499 | 0.032616 |
| NM_175324 | Acad11 | −1.65447 | 0.002518 | 0.032734 |
| NM_010591 | Jun | 1.703513 | 0.002522 | 0.032751 |
| NM_008295 | Hsd3b5 | −3.11398 | 0.002537 | 0.032852 |
| NM_031163 | Col2a1 | 1.506431 | 0.002597 | 0.033354 |
| NM_133894 | Ugt2b38 | −1.50103 | 0.002636 | 0.033739 |
| X71478 | Cyp4a10 | −1.63295 | 0.002697 | 0.034276 |
| NM_176963 | Galm | −1.6013 | 0.002723 | 0.03455 |
| NR_003640 | Rnf138rt1 | 1.620201 | 0.002744 | 0.03468 |
| NM_177741 | Ppp1r3b | −1.63394 | 0.002754 | 0.034744 |
| ENSMUST00000055994 | D830014E11Rik | 1.692737 | 0.002754 | 0.034744 |
| NM_023646 | Dnaja3 | −1.50856 | 0.002771 | 0.034904 |
| NM_008676 | Nbr1 | −1.7276 | 0.002794 | 0.035096 |
| NM_021491 | Smpd3 | 1.513757 | 0.002812 | 0.035212 |
| NM_138313 | Bmf | −1.91629 | 0.002821 | 0.035274 |
| NM_001042591 | Arrdc3 | −1.59572 | 0.002829 | 0.035311 |
| NM_023624 | Lrat | 1.525363 | 0.002852 | 0.035521 |
| NM_011693 | Vcam1 | 1.587902 | 0.002893 | 0.035915 |
| NM_013743 | Pdk4 | 1.649625 | 0.002932 | 0.03624 |
| NM_007742 | Col1a1 | 1.602544 | 0.002939 | 0.036278 |
| NM_007742 | Col1a1 | 1.612292 | 0.002977 | 0.036592 |
| NM_028227 | Brap | −1.6422 | 0.002981 | 0.036629 |
| NM_031163 | Col2a1 | 1.504739 | 0.003051 | 0.037168 |
| NM_009997 | Cyp2a4 | −1.74885 | 0.003057 | 0.037218 |
| NM_011393 | Slc1a2 | −1.49971 | 0.003062 | 0.037258 |
| NM_009205 | Slc3a1 | 1.532392 | 0.00311 | 0.037612 |
| NM_011394 | Slc20a2 | −1.70146 | 0.003142 | 0.037816 |
| NM_011254 | Rbp1 | 1.840969 | 0.003197 | 0.038213 |
| NM_010591 | Jun | 1.681428 | 0.003211 | 0.038313 |
| NM_010591 | Jun | 1.648451 | 0.003257 | 0.038651 |
| NM_144803 | Chrna2 | −1.57794 | 0.003308 | 0.039064 |
| NM_009503 | Vcp | −1.56431 | 0.003329 | 0.039184 |
| NM_001009927 | Smcr7 | −1.49907 | 0.003383 | 0.039667 |
| NM_007742 | Col1a1 | 1.586017 | 0.003425 | 0.039953 |
| NM_007468 | Apoa4 | 2.002251 | 0.00344 | 0.040015 |
| NM_021539 | Wsb2 | −1.53436 | 0.003477 | 0.040276 |

**Table S4.** *Cont.*

| **SystematicName** | **GeneSymbol** | **Fold Change** | ***p* Value** | **FDR** |
| --- | --- | --- | --- | --- |
| NM_053083 | Loxl4 | 1.50682 | 0.003617 | 0.041316 |
| NR_003513 | Neat1 | 1.672094 | 0.003684 | 0.041722 |
| NM_033074 | Tars | −1.5726 | 0.003698 | 0.041771 |
| NAP014889-001 | NA | −1.58031 | 0.003741 | 0.042093 |
| NM_015760 | Nox4 | −1.55043 | 0.003748 | 0.042101 |
| NM_007489 | Arntl | 1.751649 | 0.003749 | 0.042101 |
| NM_019696 | Cpxm1 | 1.538822 | 0.003797 | 0.042417 |
| NM_011999 | Clec4a2 | 1.657416 | 0.003845 | 0.042761 |
| NM_011594 | Timp2 | 1.630613 | 0.003956 | 0.043622 |
| NM_010591 | Jun | 1.656171 | 0.003959 | 0.043637 |
| NM_008796 | Pctp | −1.78952 | 0.003994 | 0.043891 |
| NM_177445 | Dars | −1.67983 | 0.004055 | 0.044309 |
| NR_003278 | Pigt | 1.765527 | 0.004087 | 0.044588 |
| NM_010591 | Jun | 1.659546 | 0.004186 | 0.045199 |
| NM_016720 | Neu3 | 1.579732 | 0.004187 | 0.045199 |
| NM_010390 | H2-Q1 | 1.501371 | 0.004213 | 0.04546 |
| NM_144856 | Slc22a7 | −1.61492 | 0.004267 | 0.045839 |
| NM_008548 | Man1a | −1.7415 | 0.004386 | 0.04662 |
| NM_146718 | Olfr430 | −1.52622 | 0.004424 | 0.046898 |
| NM_009264 | Sprr1a | 1.555669 | 0.004429 | 0.046898 |
| NM_007640 | Cd1d2 | −1.77267 | 0.004483 | 0.047265 |
| NM_172498 | Ptk2b | −1.714 | 0.004484 | 0.047269 |
| NM_175093 | Trib3 | −1.52069 | 0.004682 | 0.048526 |
| NM_021472 | Rnase4 | −1.61616 | 0.004716 | 0.048742 |
| NR_028574 | Snhg8 | 1.537478 | 0.004721 | 0.048786 |
| NM_001033293 | Uap1l1 | 1.571843 | 0.004746 | 0.04892 |
| NM_020568 | Plin4 | 1.607004 | 0.004759 | 0.048996 |
| NM_011862 | Pacsin2 | −1.53495 | 0.004815 | 0.049438 |
| NM_010591 | Jun | 1.647952 | 0.004865 | 0.049794 |

Positive fold change = Higher expression in the LH group. Negative fold change = Higher expression in the HH group.

**Table S5.** Gene set enrichment analysis for liver of HH offspring compared with HL offspring.

| **PathID** | **Pathway** | **NGenes** | **PropDown** | **PropUp** | **Direction** | ***p* Value** |
| --- | --- | --- | --- | --- | --- | --- |
| 4810861 | Zinc transporters | 27 | 0.259259 | 0 | Down | 0.001998 |
| 4809904 | Defective AMN causes hereditary megaloblastic anemia 1 | 123 | 0.227642 | 0.04878 | Down | 0.001998 |
| 4810939 | Nonsense Mediated Decay Independent of the Exon Junction Complex | 131 | 0.022901 | 0.435115 | Up | 0.003996 |
| 4809903 | Defective LMBRD1 causes methylmalonic aciduria and homocystinuria type cblF | 123 | 0.227642 | 0.04878 | Down | 0.003996 |
| 4809905 | Defective CUBN causes hereditary megaloblastic anemia 1 | 123 | 0.227642 | 0.04878 | Down | 0.003996 |
| 4810836 | Sphingolipid de novo biosynthesis | 58 | 0.12069 | 0.017241 | Down | 0.003996 |
| 4810940 | Nonsense-Mediated Decay | 169 | 0.023669 | 0.337278 | Up | 0.005994 |
| 4809947 | GTP hydrolysis and joining of the 60S ribosomal subunit | 172 | 0.052326 | 0.343023 | Up | 0.005994 |
| 4810902 | Nucleosome assembly | 55 | 0.036364 | 0.254545 | Up | 0.005994 |
| 4809906 | Defective MTRR causes methylmalonic aciduria and homocystinuria type cblE | 123 | 0.227642 | 0.04878 | Down | 0.005994 |
| 4809896 | Metabolism of water-soluble vitamins and cofactors | 123 | 0.227642 | 0.04878 | Down | 0.005994 |
| 4810390 | Cytosolic sensors of pathogen-associated DNA | 92 | 0.054348 | 0.163043 | Up | 0.005994 |
| 4810291 | 3' -UTR-mediated translational regulation | 166 | 0.054217 | 0.349398 | Up | 0.007992 |
| 4809952 | Ribosomal scanning and start codon recognition | 106 | 0.075472 | 0.292453 | Up | 0.007992 |
| 912497 | Meiotic Recombination | 80 | 0 | 0.2875 | Up | 0.007992 |
| 4809916 | Defective HLCS causes multiple carboxylase deficiency | 123 | 0.227642 | 0.04878 | Down | 0.007992 |
| 4809898 | Defective TCN2 causes hereditary megaloblastic anemia | 123 | 0.227642 | 0.04878 | Down | 0.007992 |
| 4809910 | Defective MMACHC causes methylmalonic aciduria and homocystinuria type cblC | 123 | 0.227642 | 0.04878 | Down | 0.007992 |
| 4809913 | Defective CD320 causes methylmalonic aciduria | 123 | 0.227642 | 0.04878 | Down | 0.007992 |
| 4809907 | Defective MTR causes methylmalonic aciduria and homocystinuria type cblG | 123 | 0.227642 | 0.04878 | Down | 0.007992 |
| 4810614 | FCERI mediated NF-kB activation | 64 | 0.015625 | 0.171875 | Up | 0.007992 |
| 4810293 | Eukaryotic Translation Elongation | 130 | 0.015385 | 0.438462 | Up | 0.00999 |
| 4809956 | Formation of a pool of free 40S subunits | 141 | 0.021277 | 0.404255 | Up | 0.00999 |
| 4810290 | L13a-mediated translational silencing of Ceruloplasmin expression | 166 | 0.054217 | 0.349398 | Up | 0.00999 |
| 4809948 | Cap-dependent Translation Initiation | 182 | 0.054945 | 0.32967 | Up | 0.00999 |
| 4809899 | Defects in cobalamin (B12) metabolism | 123 | 0.227642 | 0.04878 | Down | 0.00999 |
| 4809900 | Defects in vitamin and cofactor metabolism | 123 | 0.227642 | 0.04878 | Down | 0.00999 |
| 4809902 | Defective GIF causes intrinsic factor deficiency | 123 | 0.227642 | 0.04878 | Down | 0.00999 |

**Table S5.** *Cont.*

| **PathID** | **Pathway** | **NGenes** | **PropDown** | **PropUp** | **Direction** | ***p* Value** |
| --- | --- | --- | --- | --- | --- | --- |
| 4809908 | Defective MMAB causes methylmalonic aciduria type cblB | 123 | 0.227642 | 0.04878 | Down | 0.00999 |
| 4809911 | Defective MMAA causes methylmalonic aciduria type cblA | 123 | 0.227642 | 0.04878 | Down | 0.00999 |
| 4810221 | Elongation arrest and recovery | 50 | 0.02 | 0.22 | Up | 0.00999 |

NGenes = Number of genes in pathway; PropUP = Proportion of genes UP (Higher in first group); PropDOWN = Proportions of genes DOWN (Lower in first group).

**Table S6.** Genes differentially expressed in the liver of offspring in the HH group compared with those in the HL group.

| **SystematicName** | **GeneSymbol** | **Fold Change** | ***p* Value** | **FDR** |
| --- | --- | --- | --- | --- |
| NM_172838 | Slc16a12 | −1.71865 | 9.95E-08 | 0.001439 |
| NM_010197 | Fgf1 | −1.60602 | 3.66E-07 | 0.003171 |
| ENSMUST00000102977 | Hist1h4i | 1.653202 | 4.42E-07 | 0.003194 |
| NM_206537 | Cyp2c54 | −2.7243 | 1.37E-06 | 0.00659 |
| NM_146948 | Olfr342 | −1.58677 | 1.90E-06 | 0.006801 |
| NM_001039354 | Lin7a | −1.68677 | 2.04E-06 | 0.006801 |
| NM_145713 | Hist1h1d | 1.592263 | 2.44E-06 | 0.007104 |
| NM_145364 | Akr1d1 | −1.90736 | 3.18E-06 | 0.007249 |
| NM_007900 | Ect2 | 1.510253 | 4.54E-06 | 0.009385 |
| NM_016919 | Col5a3 | 1.621198 | 4.90E-06 | 0.009659 |
| NM_027881 | Osbpl3 | 1.647251 | 5.71E-06 | 0.010328 |
| NM_028696 | Nabp1 | 1.693552 | 6.93E-06 | 0.010635 |
| NM_001025566 | Chka | 1.789656 | 7.06E-06 | 0.010635 |
| NM_011387 | Slc10a1 | −1.75192 | 7.90E-06 | 0.010758 |
| NM_007812 | Cyp2a5 | −2.09996 | 8.00E-06 | 0.010758 |
| NM_010197 | Fgf1 | −1.51287 | 8.46E-06 | 0.010758 |
| NM_153484 | Tef | −1.62703 | 9.74E-06 | 0.011002 |
| NM_146316 | Olfr726 | −1.54924 | 1.10E-05 | 0.011671 |
| NM_011817 | Gadd45g | 1.778807 | 1.50E-05 | 0.013736 |
| NM_133903 | Spon2 | 1.937886 | 1.66E-05 | 0.013736 |
| ENSMUST00000131882 | Cpn1 | 1.582261 | 1.68E-05 | 0.013736 |
| NM_011267 | Rgs16 | −3.9352 | 1.83E-05 | 0.014161 |
| NM_010001 | Cyp2c37 | −2.43115 | 2.80E-05 | 0.01678 |
| NM_001033302 | Gm129 | −3.07786 | 2.83E-05 | 0.01678 |
| ENSMUST00000124229 | Prkd3 | −1.58141 | 2.86E-05 | 0.01678 |
| NM_153484 | Tef | −2.30822 | 2.92E-05 | 0.016893 |
| NM_198092 | Usp2 | −2.21454 | 3.65E-05 | 0.018106 |
| NM_009997 | Cyp2a4 | −1.88074 | 4.08E-05 | 0.018588 |
| NM_145134 | Spsb4 | 1.60403 | 4.11E-05 | 0.018588 |
| NM_017379 | Tuba8 | 1.85727 | 4.29E-05 | 0.019186 |
| NM_010444 | Nr4a1 | 1.902105 | 4.78E-05 | 0.019958 |
| NM_133903 | Spon2 | 2.015275 | 5.34E-05 | 0.021852 |
| NM_175111 | Hspbap1 | 1.558344 | 5.41E-05 | 0.021947 |
| NR_029475 | 4930581F22Rik | −1.52536 | 5.53E-05 | 0.022215 |
| ENSMUST00000125411 | Chka | 3.13363 | 6.04E-05 | 0.023324 |
| NM_019792 | Cyp3a25 | −1.6453 | 7.86E-05 | 0.02644 |
| NM_022331 | Herpud1 | −1.76483 | 8.10E-05 | 0.02702 |
| NM_007669 | Cdkn1a | 1.72696 | 9.31E-05 | 0.029902 |
| NR_030711 | Mir22hg | −1.51728 | 0.000101 | 0.031394 |
| NM_010591 | Jun | 2.074359 | 0.000123 | 0.034849 |
| NM_001042591 | Arrdc3 | −1.85485 | 0.000133 | 0.035681 |
| NM_007815 | Cyp2c29 | -1.58334 | 0.000143 | 0.036336 |
| NM_009997 | Cyp2a4 | −2.09496 | 0.000148 | 0.036336 |

**Table S6.** *Cont.*

| **SystematicName** | **GeneSymbol** | **Fold Change** | ***p* Value** | **FDR** |
| --- | --- | --- | --- | --- |
| NM_007669 | Cdkn1a | 1.7668 | 0.000156 | 0.036701 |
| NM_007669 | Cdkn1a | 1.716589 | 0.000159 | 0.036701 |
| NM_008748 | Dusp8 | 1.523644 | 0.00016 | 0.036701 |
| NM_021456 | Ces1g | −1.60026 | 0.000163 | 0.036701 |
| NM_201645 | Ugt1a1 | −1.98232 | 0.000167 | 0.036792 |
| NM_021456 | Ces1g | −1.58319 | 0.000177 | 0.036792 |
| NM_007669 | Cdkn1a | 1.742766 | 0.00018 | 0.036792 |
| NM_153800 | Arhgap22 | 1.55679 | 0.000181 | 0.036792 |
| NM_178373 | Cidec | 2.971139 | 0.000182 | 0.036792 |
| NM_134164 | Syt12 | 1.561944 | 0.000184 | 0.036792 |
| NM_001080924 | NA | −1.50822 | 0.000187 | 0.037048 |
| NM_053110 | Gpnmb | 2.648858 | 0.000194 | 0.037064 |
| NM_016974 | Dbp | −4.59049 | 0.000204 | 0.037494 |
| NM_007669 | Cdkn1a | 1.69474 | 0.000211 | 0.037541 |
| NM_010591 | Jun | 1.952118 | 0.000212 | 0.037541 |
| NM_172928 | Dclk3 | −1.5418 | 0.000213 | 0.037574 |
| NM_144845 | Ugt3a2 | −1.54066 | 0.000214 | 0.037574 |
| NM_010591 | Jun | 1.909101 | 0.000216 | 0.03786 |
| NM_001184981 | NA | −2.22496 | 0.00023 | 0.038789 |
| NM_031197 | Slc2a2 | −1.94156 | 0.000232 | 0.038841 |
| NM_015786 | Hist1h1c | 1.668466 | 0.000236 | 0.03939 |
| NM_027249 | Tlcd2 | −1.58777 | 0.000237 | 0.039396 |
| NM_007669 | Cdkn1a | 1.697698 | 0.000239 | 0.039396 |
| NM_011575 | Tff3 | 1.585642 | 0.000267 | 0.041418 |
| NM_010212 | Fhl2 | 1.809615 | 0.000269 | 0.041418 |
| NM_153392 | Ttc39a | 1.558003 | 0.000273 | 0.041661 |
| NM_020568 | Plin4 | 1.877393 | 0.000275 | 0.041852 |
| NM_007669 | Cdkn1a | 1.722439 | 0.000283 | 0.042124 |
| NM_007669 | Cdkn1a | 1.661303 | 0.000289 | 0.042124 |
| NR_028575 | 0610007N19Rik | 1.517116 | 0.000289 | 0.042124 |
| NM_019641 | Stmn1 | 1.571763 | 0.000291 | 0.042157 |
| NM_028113 | Amer2 | −1.58131 | 0.000305 | 0.04253 |
| NM_007669 | Cdkn1a | 1.710128 | 0.000318 | 0.042793 |
| NM_001164885 | Lpin2 | −1.76394 | 0.000325 | 0.042793 |
| NM_010591 | Jun | 1.920333 | 0.000328 | 0.042793 |
| NM_146245 | Lrit1 | −1.53825 | 0.000331 | 0.042793 |
| NM_011414 | Slpi | 1.947288 | 0.000352 | 0.044016 |
| NM_011448 | Sox9 | 1.626702 | 0.000355 | 0.044016 |
| NM_021366 | Klf13 | −1.53406 | 0.00036 | 0.044016 |
| NM_007742 | Col1a1 | 1.73359 | 0.000364 | 0.044016 |
| NM_010591 | Jun | 1.897396 | 0.000372 | 0.044236 |
| NM_011448 | Sox9 | 1.520081 | 0.000374 | 0.044236 |
| NM_010591 | Jun | 1.911433 | 0.000377 | 0.044236 |
| NM_011584 | Nr1d2 | −1.80201 | 0.000381 | 0.044391 |
| NM_015763 | Lpin1 | −2.14542 | 0.000394 | 0.045018 |

**Table S6.** *Cont.*

| **SystematicName** | **GeneSymbol** | **Fold Change** | ***p* Value** | **FDR** |
| --- | --- | --- | --- | --- |
| NM_007618 | Serpina6 | −1.6965 | 0.000413 | 0.045312 |
| NM_010591 | Jun | 1.888236 | 0.000418 | 0.045312 |
| NM_007559 | Bmp8b | 1.64417 | 0.000421 | 0.045312 |
| NM_025557 | Pcp4l1 | −1.63075 | 0.000422 | 0.045312 |
| NM_013490 | Chka | 1.972215 | 0.000432 | 0.045941 |
| NM_018869 | Grk5 | 1.561774 | 0.000434 | 0.045941 |
| NM_007403 | Adam8 | 1.73698 | 0.000437 | 0.045941 |
| NM_016854 | Ppp1r3c | 1.764045 | 0.000441 | 0.045999 |
| NM_007742 | Col1a1 | 1.733388 | 0.000452 | 0.046318 |
| NM_010591 | Jun | 1.866188 | 0.000455 | 0.046318 |
| NM_009306 | Syt1 | −1.62056 | 0.000463 | 0.046934 |
| NM_023223 | Cdc20 | 1.793985 | 0.000472 | 0.047298 |
| ENSMUST00000128428 | Frmd5 | 1.682318 | 0.000475 | 0.047376 |
| NM_133898 | N4bp2l1 | −1.69547 | 0.00048 | 0.047716 |
| NM_145434 | Nr1d1 | −1.65024 | 0.000494 | 0.048567 |
| NM_007669 | Cdkn1a | 1.636992 | 0.000505 | 0.049265 |

Positive fold change = Higher expression in the HH group. Negative fold change = Higher expression in the HL group.

**Table S7.** Gene set enrichment analysis for colon of HH offspring compared with LH offspring.

| **PathID** | **Pathway** | **NGenes** | **PropDown** | **PropUp** | **Direction** | ***p* Value** |
| --- | --- | --- | --- | --- | --- | --- |
| 4809824 | G1/S Transition | 168 | 0.02381 | 0.261905 | Up | 0.001998 |
| 4810996 | Mitochondrial Protein Import | 73 | 0 | 0.315068 | Up | 0.001998 |
| 4810438 | Cyclin A:Cdk2-associated events at S phase entry | 101 | 0.019802 | 0.168317 | Up | 0.001998 |
| 4810881 | Regulation of mRNA Stability by Proteins that Bind AU-rich Elements | 140 | 0.057143 | 0.142857 | Up | 0.001998 |
| 4809875 | The citric acid (TCA) cycle and respiratory electron transport | 177 | 0.028249 | 0.338983 | Up | 0.003996 |
| 4810682 | Amine compound SLC transporters | 46 | 0.282609 | 0 | Down | 0.003996 |
| 4810485 | SCF(Skp2)-mediated degradation of p27/p21 | 83 | 0 | 0.204819 | Up | 0.003996 |
| 4809836 | Cyclin E associated events during G1/S transition | 99 | 0.020202 | 0.171717 | Up | 0.003996 |
| 4810912 | HS-GAG biosynthesis | 43 | 0.093023 | 0.023256 | Down | 0.003996 |
| 4810705 | Other semaphorin interactions | 42 | 0.238095 | 0.047619 | Down | 0.005994 |
| 4809825 | Mitotic G1-G1/S phases | 211 | 0.033175 | 0.246445 | Up | 0.005994 |
| 4809841 | p53-Dependent G1/S DNA damage checkpoint | 89 | 0.022472 | 0.157303 | Up | 0.005994 |
| 4809842 | G1/S DNA Damage Checkpoints | 93 | 0.021505 | 0.182796 | Up | 0.007992 |
| 4809821 | Synthesis of DNA | 148 | 0.02027 | 0.25 | Up | 0.00999 |
| 4809928 | Metabolism of nucleotides | 127 | 0.015748 | 0.23622 | Up | 0.00999 |
| 4809813 | Cell Cycle | 869 | 0.051784 | 0.218642 | Up | 0.00999 |
| 4810117 | RNA Polymerase III Transcription Initiation From Type 2 Promoter | 44 | 0.068182 | 0.136364 | Up | 0.00999 |
| 4809827 | Orc1 removal from chromatin | 110 | 0.027273 | 0.209091 | Up | 0.00999 |
| 4811003 | Voltage gated Potassium channels | 49 | 0.163265 | 0 | Down | 0.00999 |
| 4811175 | TRP channels | 41 | 0.170732 | 0.02439 | Down | 0.00999 |
| 4811051 | Chondroitin sulfate/dermatan sulfate metabolism | 73 | 0.082192 | 0.027397 | Down | 0.00999 |

NGenes = Number of genes in pathway. PropUP = Proportion of genes UP (Higher in first group). PropDOWN = Proportions of genes DOWN (Lower in first group).
